# Bayesian optimization for uncertainty-aware prediction of rainfall-induced deformation in embankment dams

**DOI:** 10.1038/s41598-026-46994-w

**Published:** 2026-04-14

**Authors:** Mohammed Nasser, Eleyas Assefa, Siraj M. Assefa, Teshome B. Kebede, Constantinos C. Sachpazis, Lysandros Pantelidis

**Affiliations:** 1 Department of Civil Engineering, College of Engineering, Addis Ababa Science and Technology University, Addis Ababa, Ethiopia; 2 Independent Consultant, Atlanta, USA; 3 Geotechnical and Mining Engineering Division, University of Western Macedonia, Kozani, Greece; 4 Department of Civil Engineering and Geomatics, Cyprus University of Technology, Limassol, Cyprus

**Keywords:** Engineering, Hydrology, Natural hazards

## Abstract

Reliable early warning of embankment dam failure requires predictive models that are accurate, physically consistent, and uncertainty-calibrated. This study proposes a hybrid physics-informed Bayesian deep learning framework integrating coupled u-p Biot consolidation-based finite element modeling (OpenSeesPy) with an ANN-LSTM-MDN architecture optimized via Bayesian Optimization. Deterministic hydro-mechanical responses provide physically grounded descriptors and regularization targets, while the probabilistic network decomposes uncertainty into epistemic and aleatory components. Physics-informed penalty terms enforce consolidation-consistent behavior. The approach introduces adaptive, composition-dependent uncertainty scaling to account for heterogeneous borrow materials and non-stationary rainfall effect. A novel Uncertainty Calibration Score (UCS) jointly optimizes predictive sharpness and empirical coverage. Material-adaptive dropout rates further regularize predictions for variable soil compositions. Validation on construction-phase monitoring data from the Megech Dam demonstrates substantial improvements: Negative Log-Likelihood decreased from − 2.36 to − 2.52, CRPS decreased by 33.7% ($0.092 \to 0.061$), and PICP increased from 0.86 to 0.93. Epistemic uncertainty reduced by 37.7%, while aleatoric variability remained captured. Adaptive prediction intervals revealed a pre-failure shift, with epistemic uncertainty rising to ~ 72% of total variance 8–12 weeks before observed failure. Statistical validation via block-bootstrap resampling, paired hypothesis testing (*p *< 0.0001), and ten-fold stratified cross-validation (CV < 8%) confirms significance and stability. This framework advances embankment dam forecasting by coupling geotechnical physics with Bayesian deep learning, providing reproducible, interpretable, and uncertainty-aware early warning insights for construction-phase variability.

## Introduction

Rainfall-induced deformation in embankment dams is particularly critical during construction, when incompletely compacted and partially saturated fills are highly sensitive to pore-pressure increases^1^. Rainfall infiltration combined with staged construction loading can trigger delayed consolidation and creep that may progressively evolve toward failure^2^. The collapse of Megech Dam illustrates this mechanism: occurring during construction without reservoir impoundment, rainfall-driven wetting led from gradual creep and tension cracking to a final 24-m translational slide^3^, demonstrating the nonlinear interaction of hydro-mechanical and climatic processes in triggering progressive instability.

Our earlier deterministic study used a physics-informed hybrid FEM–ANN–LSTM framework combining coupled hydro-mechanical FEM simulations, monitoring data, and PCA-based rainfall indices^4^. It achieved strong performance (R^2^ = 0.94) and revealed a 2–3-week lag between rainfall peaks and pore-pressure buildup. However, it produced only point estimates, limiting early-warning reliability where confidence bounds are required^5^. The December 2021 Megech failure highlighted that point forecasts alone are insufficient for risk-informed decisions under sparse or noisy monitoring conditions.

To address this gap, the deterministic model was extended to a probabilistic deep-sequence framework integrating Monte Carlo Dropout, Mixture Density Networks, and FEM constraints^6^. The model showed strong calibration (PICP ≈ 95%, CRPS = 0.24). Notably, epistemic uncertainty rose from 39 to 72% within 8–12 weeks before failure, coinciding with crack development and wetting on site^6^. This indicates probabilistic deep learning can detect regime shifts toward instability earlier than deterministic models, which often struggle with time-dependent infiltration and partial saturation during construction^5^.

A major challenge is calibrating hybrid uncertainty-aware models under non-stationary conditions. Poorly tuned dropout rates, MDN widths, or physics weights can cause overconfident, miscalibrated predictions. LSTMs capture temporal patterns but may produce physically inconsistent forecasts or fail to generalize spatially under extremes^7^. Physics-informed hybrids mitigate this but can still fail if constraints are weak^7^. MDNs capture aleatory uncertainty but may assign high likelihood to unrealistic modes without physical context^8^, while deep learning rainfall–runoff models can break down under unprecedented hydrological extremes^9^.

Physics-informed machine learning improves physical consistency but often struggles to learn long-term history dependence and can create highly nonconvex training landscapes^10^. In dam-break modeling, PINNs also face training difficulties with steep gradients or discontinuities, often requiring hybridization with numerical simulations^11^. Moreover, inverse identification of hydraulic parameters in earth dams remains ill-posed without strong regularization, especially when monitoring data are sparse or noisy during construction^12^.

Complementary machine learning methods have been applied in geotechnical stability forecasting, each with strengths and limits. Support vector machines and random forests predict slope stability but lack temporal memory, missing sequential rainfall–deformation effects^13^. Gaussian process regression provides uncertainty quantification but struggles with long time-series typical of dam monitoring^14^. Time-series CNNs aid anomaly detection but may miss evolving lag structures, while ensembles improve robustness yet offer limited insight into whether predictive confidence arises from data density or model design.

Bayesian neural networks provide principled uncertainty quantification via variational inference or MCMC, but traditional BNNs are computationally heavy for high-dimensional, long-sequence problems, limiting real-time early-warning use. Advances in scalable variational inference and stochastic gradient MCMC improve tractability, though applications to multi-physics embankment problems remain limited^15^. Sequential Monte Carlo methods show promise for state-space geotechnical inverse problems, enabling real-time parameter updates, but remain computationally intensive for operational deployment.

Bayesian Optimization offers a principled approach for hyperparameter tuning, using probabilistic surrogate models to explore high-dimensional spaces while balancing accuracy and uncertainty^16^. In geotechnical applications, it improves machine learning performance for soil parameter inversion and settlement prediction, efficiently leveraging prior knowledge^17^. Bayesian optimization has also enabled probabilistic slope-failure back-analysis with 60% fewer FEM evaluations^18^ and enhanced relevance vector machine models for liquefaction assessment, yielding 15–20% higher classification accuracy with well-calibrated uncertainty.

Automated calibration is particularly valuable for embankment dams, where soil heterogeneity, construction variability, climate extremes, and sequencing effects interact in non-stationary ways. Bayesian Optimization enables (i) material-driven regularization that scales epistemic uncertainty using Mahalanobis distance from known borrow materials, (ii) adaptive prediction intervals based on epistemic–aleatory composition, (iii) physics-informed objectives enforcing Terzaghi-consistent settlement constraints, and (iv) convergence guided by an Uncertainty Calibration Score balancing coverage and sharpness. Complementary probabilistic approaches, including Bayesian neural networks and physics-informed autoencoders, also show promise for geotechnical inverse problems and constitutive calibration, achieving accurate soil parameter estimation with fewer experiments while preserving physical consistency^19^.

Despite progress, key gaps remain. First, most embankment monitoring frameworks do not explicitly separate epistemic and aleatory uncertainty, limiting interpretability for risk decisions. Second, physics-informed regularization often relies on weak loss penalties rather than hard constraints, allowing physically inconsistent extrapolation. Third, adaptive prediction intervals reflecting sample-specific novelty (e.g., unusual rainfall or uncharacterized materials) remain underdeveloped, with most models using uniform confidence bounds. Finally, rigorous calibration of hybrid physics–ML models under non-stationary construction conditions is largely absent, as many studies assume stationary data-generating processes.

This study presents a probabilistic forecasting framework that optimizes predictive uncertainty in a hybrid FEM–ANN–LSTM–MDN model using Bayesian Optimization and physics-based regularization. Building on earlier work^4,6^, epistemic and aleatory uncertainties are explicitly separated and constrained through a physics-informed loss enforcing Terzaghi-consistent settlement. Adaptive prediction intervals scale uncertainty based on epistemic–aleatory dominance. The framework is validated using the Megech Dam failure case, advancing early-warning systems from deterministic forecasts to calibrated probabilistic predictions.

## Study area

### Location and project setting

**Fig. 1:**
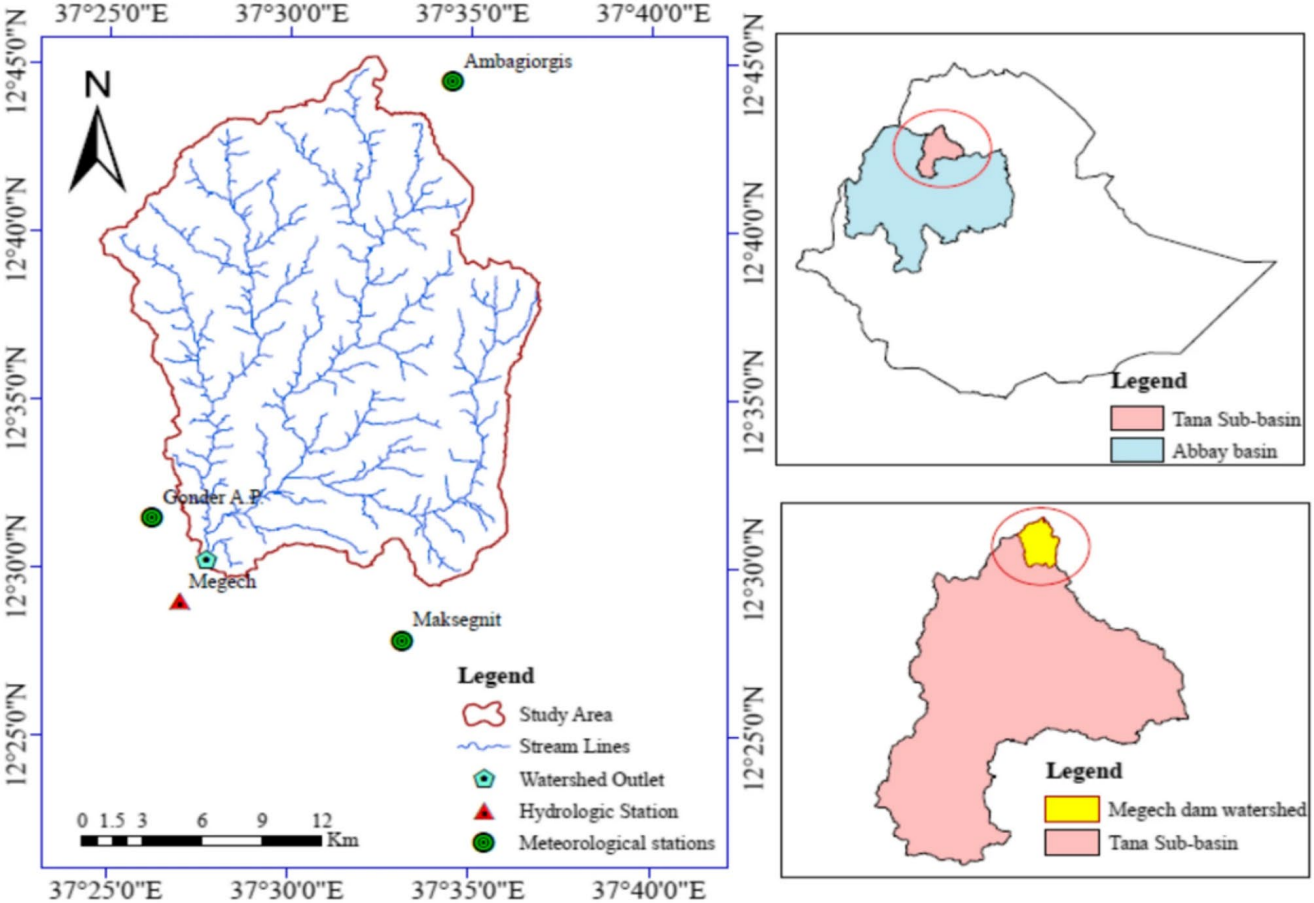
The Megech river water shed and location of the study area https://link.springer.com/article/10.1007/s13201-025-02571-6/figures/1^4^.

The Megech Dam is located in Ethiopia’s North Gondar Zone (12.62° N, 37.45° E), approximately 45 km northeast of Gondar, across the Megech River within the Lake Tana basin. The project is designed to store approximately 185 million m^3^ of water for irrigation (≈17,000 ha), municipal supply, and fisheries development^4^. The dam follows a zoned earth–rockfill configuration with a central clay core^4^ (Fig. 1).

### Construction-phase condition during failure (2020–2021)

**Fig. 2:**
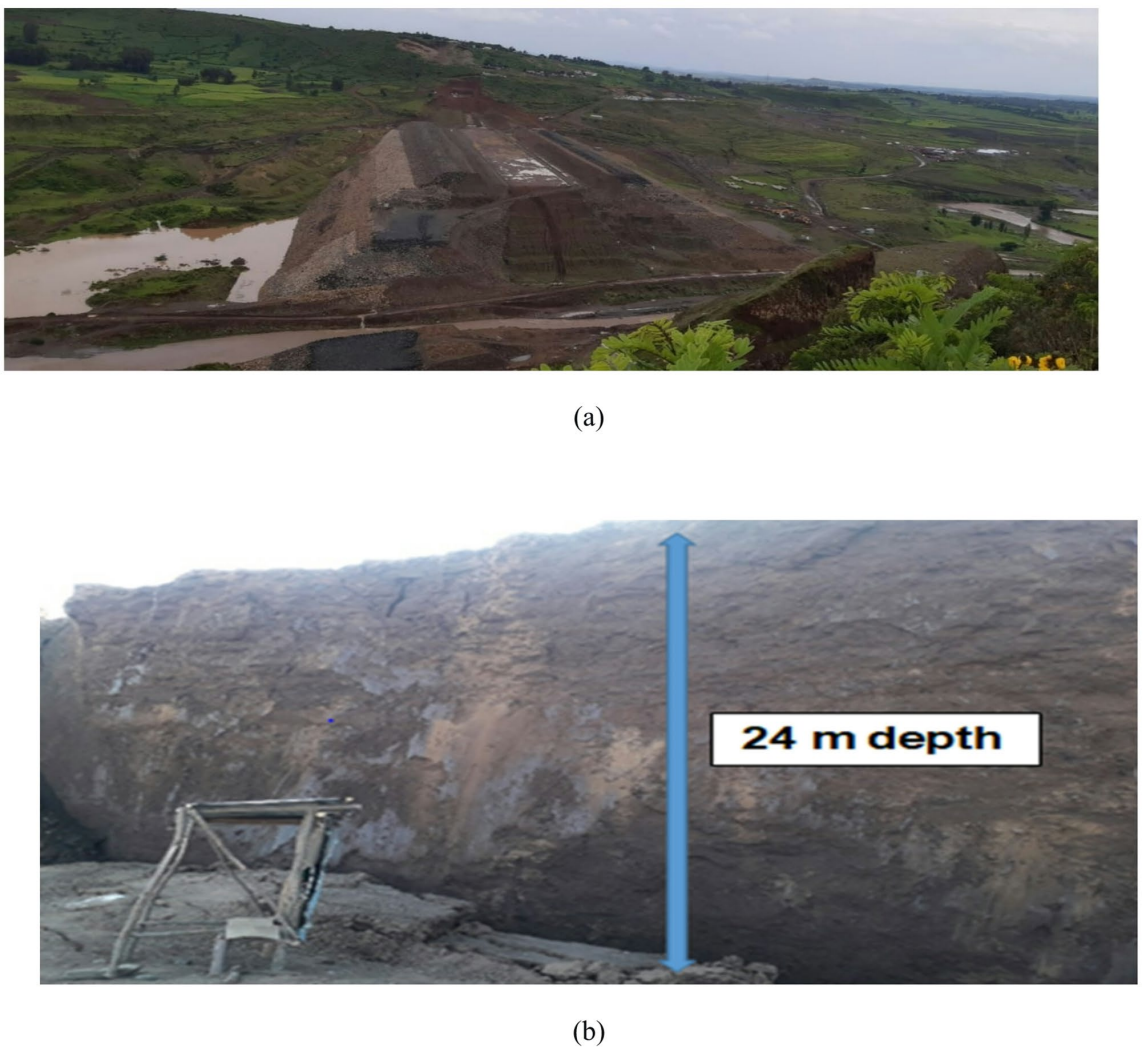
(**a**–**b**) Composite slope failures and a 24 m translational block slide observed during 2020–2021^4^.

During the 2020–2021 instability events, the reservoir had not been impounded. Only 500 m of the planned 890 m crest had been completed, leaving a 390 m opening to maintain river flow^4^. The completed section (chainage 0 + 000 to 0 + 640) had reached elevation 1930 m.a.s.l. by April 2021. As a result, hydrostatic loading from impounded water—typically a dominant stress contributor in operational dams was not present. The stress regime was therefore governed primarily by rainfall infiltration and transient seepage (Fig. 2).

### Geometry and zoning

The embankment has a maximum height of 77.1 m, crest width of 10 m, upstream slope of 1 V:2H, and downstream slope ranging from 1 V:1.75H to 1 V:2H. The total design fill volume is approximately 9.1 million m^3^. The zoned configuration (clay core, filters, transitions, and rockfill shells) is consistent with international design recommendations for seepage control and internal stability^20,21^.

### Material properties

Clay core materials sourced from five borrow areas exhibit Plasticity Index values between 22.2 and 28.6%, classified as MH–CH. Compacted permeability ranges from 10^−6^ to 10^−8^ cm/s, consistent with low-permeability core specifications for seepage control^22^. Effective shear strength parameters derived from consolidated undrained triaxial testing indicate φ′ = 14.6°–15.9° and c′ = 28–32 kPa, with maximum dry density between 1.29 and 1.32 g/cm^3^^25^ (Ethiopian Construction Design and Supervision Works Corporation, 2022).

### Hydrological conditions and failure history

The region experienced sustained above-average rainfall between 2019 and 2021 (≈1100 mm in 2019; 1350 mm in 2020; 1450 mm in 2021)^4^ (Ethiopian Construction Design and Supervision Works Corporation, 2022). Prolonged wet–dry cycles during staged construction likely promoted desiccation cracking and enhanced infiltration pathways. Rainfall-induced pore-pressure rise and rapid strength degradation are well-documented mechanisms in partially constructed embankments^23–25^.

A major translational slide occurred in December 2021 between chainage 0 + 420 and 0 + 640, involving approximately 1.25 million m^3^ of material and vertical displacement up to 24 m over four days^4^.

### Failure mechanism

Investigations identified saturation of the clay core base as the principal trigger. Infiltration occurred through rainfall-induced cracking, localized upstream ponding against the incomplete crest, abutment spring inflow, and seepage through pervious shell materials. A weak plane at elevation ~ 1873 m.a.s.l., inclined approximately 5.5°, facilitated translational sliding. The calculated saturated factor of safety was 0.91, confirming instability^4^. This mechanism aligns with classical effective stress and transient seepage theory^26,27^.

### Relevance to the present study

Because reservoir impoundment had not occurred, deformation was driven primarily by rainfall infiltration—making this case ideal for validating the proposed rainfall-induced forecasting framework^4,6^.

## Methodology

### Framework overview

**Fig. 3:**
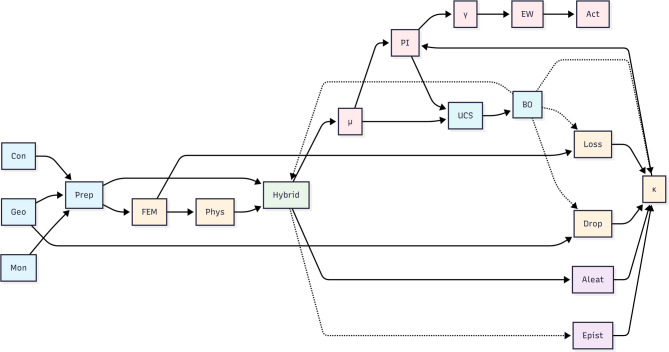
Schematic of the proposed physics-informed hybrid deep learning framework integrating multi-source monitoring data and FEM-derived descriptors with ANN, LSTM, and MDN modules to deliver probabilistic FoS forecasting, deformation prediction, and early failure warning for embankment dams.

The proposed methodology builds upon the deterministic hybrid FEM–ANN–LSTM framework previously developed and validated by the authors^4^, extending it toward uncertainty-aware forecasting. Rainfall, reservoir level, construction stage, and geotechnical properties are first processed through a coupled hydro-mechanical finite element model to generate physically consistent responses, including pore pressure and settlement. These physics-based descriptors are then combined with monitoring time-series data within a hybrid ANN–LSTM–MDN architecture, where static features are handled by an ANN, temporal dependencies by an LSTM, and aleatory uncertainty is modeled through a Mixture Density Network, while epistemic uncertainty is quantified via Monte Carlo Dropout. The overall data flow and module interactions are illustrated in Fig. 3. Bayesian Optimization calibrates architectural and regularization parameters using a physics-informed uncertainty objective.

The framework outputs mean deformation predictions, adaptive prediction intervals, and the instability index γ for uncertainty-aware early-warning assessment under non-stationary hydro-climatic conditions.

Figure 3 illustrates the complete data flow. First, monitoring data and geotechnical properties feed into the FEM model to generate physics-based descriptors. These descriptors, together with raw time series, enter the hybrid ANN–LSTM–MDN core, where the ANN processes static features, the LSTM captures temporal dependencies, the MDN models aleatory uncertainty, and Monte Carlo Dropout quantifies epistemic uncertainty. Physics-informed constraints (monotonic settlement, adaptive dropout) and adaptive prediction intervals are then applied. Finally, Bayesian Optimization tunes all hyper-parameters by maximizing the Uncertainty Calibration Score (UCS). The framework outputs mean predictions, adaptive intervals, and the instability index $\gamma$.

Although earlier exploratory versions of the framework investigated attention-based sequence models, the final architecture adopted in this study does not employ an explicit attention mechanism. Instead, temporal relevance is captured through physically informed LSTM memory, with the sequence length calibrated to the observed 8–12 week rainfall–pore-pressure–settlement lag. Uncertainty attribution and interpretability are therefore achieved through epistemic–aleatory decomposition and adaptive prediction intervals rather than attention weight visualization. Consequently, attention-weight analysis is not applicable to the finalized ANN–LSTM–MDN framework.

### FEM-based descriptor generation

Deterministic hydro–mechanical responses were generated in OpenSeesPy using the previously validated coupled consolidation model developed for the same embankment system in the adaptive multihazard study, ensuring physical consistency of the learning framework^4^. The present work directly extends that established numerical model to provide physics-based descriptors and constraints for the probabilistic ANN–LSTM–MDN forecasting framework.

A fully coupled u–p Biot formulation was implemented to simulate transient seepage, pore-pressure dissipation, and rainfall-induced settlement during staged construction. The zoned embankment and foundation were discretized with coupled displacement–pressure elements, incorporating mesh refinement in the core and monitoring zones. Effective-stress Mohr–Coulomb behavior with spatially variable permeability captured hydraulic heterogeneity. Boundary conditions included a fixed drained base, roller abutments, and time-varying upstream heads, with staged self-weight loading under nonlinear time-stepping consolidation. Extracted settlement and pore-pressure histories were used as physics-based inputs and regularization constraints in the hybrid model.

**Fig. 4:**
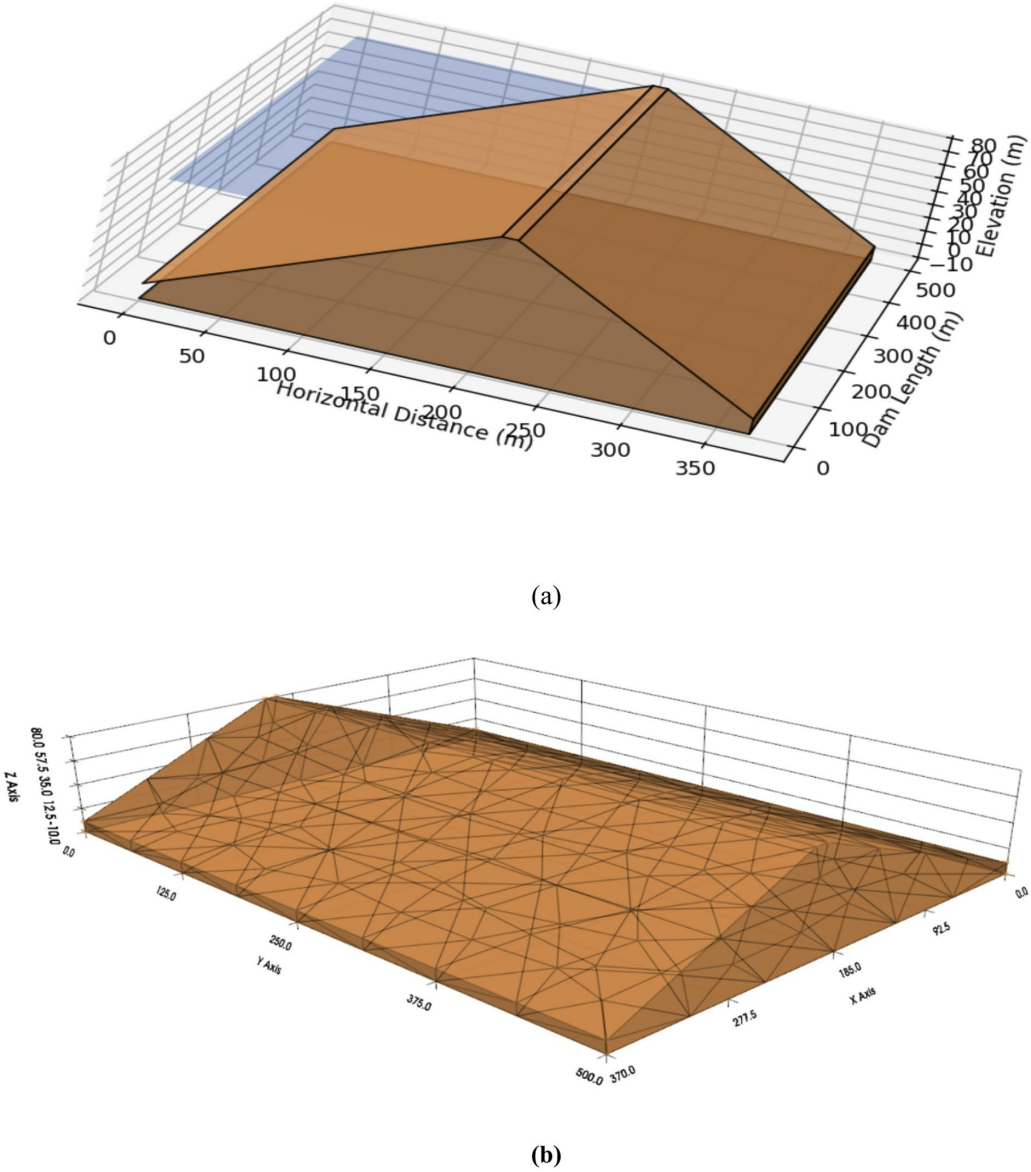
(**a**) Cross-sectional geometry and boundary conditions of the OpenSeesPy u–p consolidation model following the configuration of^4^. (**b**) The clay core, shells, drainage layers, and foundation are discretized separately. The base is fixed, abutments use roller constraints, and transient hydraulic heads representing rainfall and reservoir fluctuations are applied at the upstream face.

**Fig. 5:**
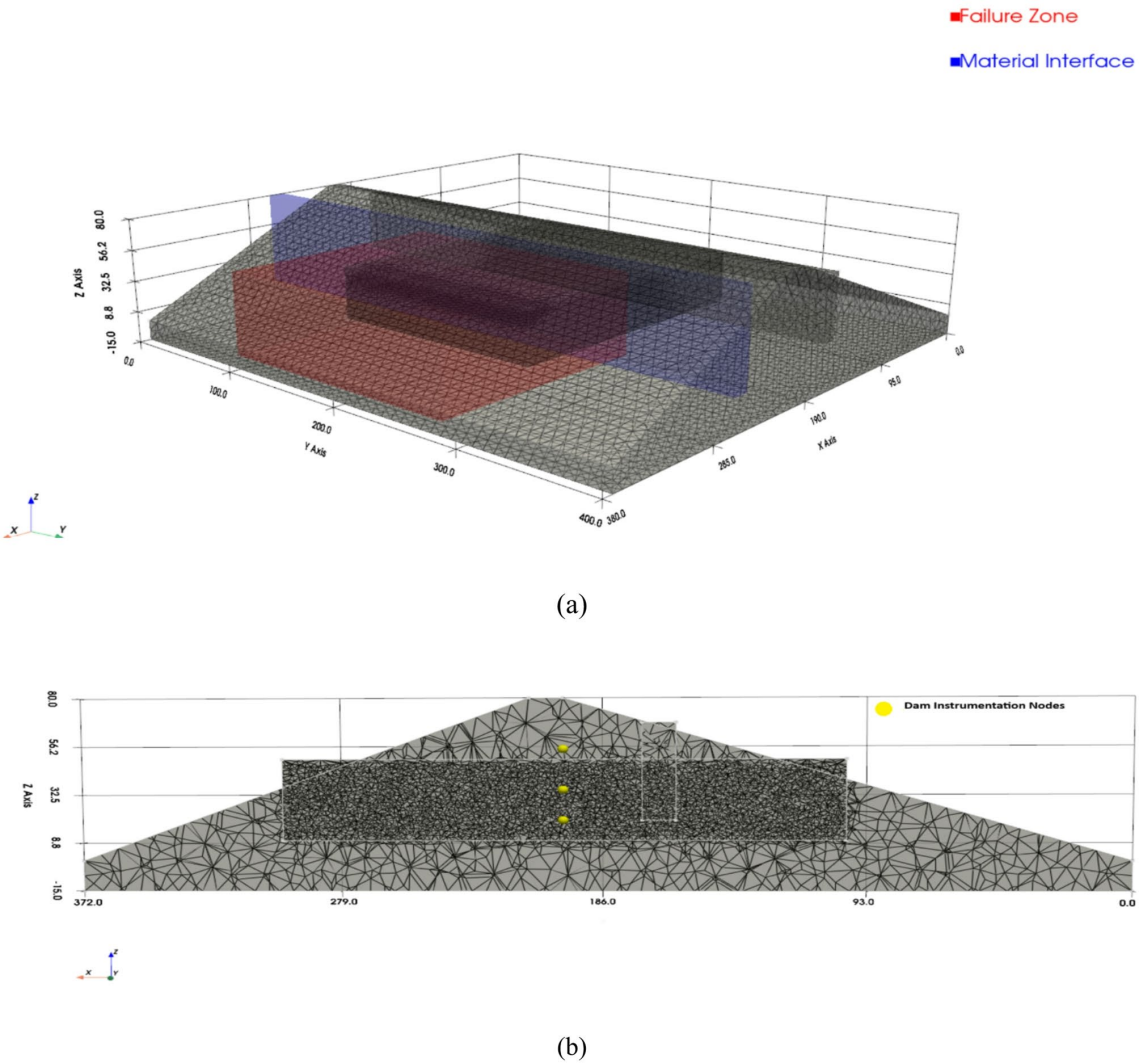
(**a**) 3D tetrahedral finite element mesh of the dam, showing refinement in the failure zone (red) and at material interfaces (blue); (**b**) Cross-section illustrating the 5.5° weak plane and saturation-front refinement in stability-critical zones. Finer elements are adopted in the clay core and around instrumentation points to capture steep pore-pressure gradients. Settlement and pore-pressure time histories extracted at these nodes provide physics-based descriptors and constraints for the hybrid ANN–LSTM–MDN framework.

The complete numerical configuration is summarized in Table 1. The model geometry, boundary conditions, and mesh discretization are illustrated in Figs. 4 and 5 to ensure reproducibility.

**Table 1:** Quantitative specification of the OpenSeesPy u–p coupled FEM model used to generate physics-based descriptors for the hybrid ANN–LSTM–MDN framework.

Category	Specification	ML descriptor
Domain	H = 80 m; ${w}_{b}=200 \mathrm{m}$; $Crest = 10 \mathrm{m}$; $L = 250 \mathrm{m}$; ${D}_{f}$ = 10 m	Geometry scaling
Mesh	$8.8k\;elems$; $9.6k\;nodes$; $28.8k\;DOF$;$38\;pts$	$u(t), p(t)$
Soil	E = 15–80 MPa; c′ = 20–35 kPa; $\varphi ^{\prime} =$ 24–32°;$\nu =$ 0.30–0.35	$\sigma ^{\prime}, \gamma$
Hydraulics	k_c_ = 10^−9^–10^−8^; k_s_ = 10^−6^–10^−5^ m/s; n ≈ 0.40	$\nabla h$
Time	$\Delta t = 1 d;$ 700–1100 steps; tol = 10^−5^	Daily sequences

### Hydro–mechanical discretization and physics-based descriptor extraction

Building on the previously validated coupled consolidation model^4^, the 80 m-high embankment (200 m base, 10 m crest, and 10 m foundation) was modeled over the 250 m critical section and discretized into ~ 8,800 *u–p* elements (~ 28,800 DOF) as detailed in Table 1. Zoned materials enabled spatially variable Mohr–Coulomb parameters and heterogeneous permeability. A fixed drained base, roller abutments, and transient rainfall-infiltration fluxes applied along the upstream slope and crest were used under nonlinear daily consolidation with staged self-weight loading. Because the failure occurred during the construction phase prior to reservoir impoundment, no reservoir head boundary condition was imposed. Mesh refinement targeted the clay core, interfaces, instrumentation nodes, the 5.5° weak plane, and the advancing saturation front. Time histories of settlement, pore pressure, and effective stress from 38 nodes (700–1,100 steps) were exported as physics-based inputs and regularization targets for the hybrid ANN–LSTM–MDN framework to resolve steep pore-pressure gradients and stress redistribution (Figs. 4 and 5).

A fixed drained base and roller abutments were used, with transient rainfall-infiltration fluxes applied along the upstream slope and crest under nonlinear daily consolidation and staged self-weight loading. Mesh refinement targeted the clay core, interfaces, instrumentation points, and the 5.5° weak plane to capture pore-pressure gradients and stress redistribution (Fig. 5).

Time histories of settlement, pore pressure, effective stress, and strain from 38 nodes (700–1,100 steps) were exported as physics-based inputs and regularization targets for the hybrid ANN–LSTM–MDN probabilistic model.

### Hybrid deep learning architecture

The deep learning architecture builds directly upon our previously validated frameworks^4,6^ extending them with adaptive mechanisms and Bayesian optimization as described in subsequent sections. As illustrated in the figure, the model processes information through two parallel branches: an Artificial Neural Network (ANN) for static, time-invariant geotechnical parameters, and a Long Short-Term Memory (LSTM) network for temporal sequences.

**Fig. 6:**
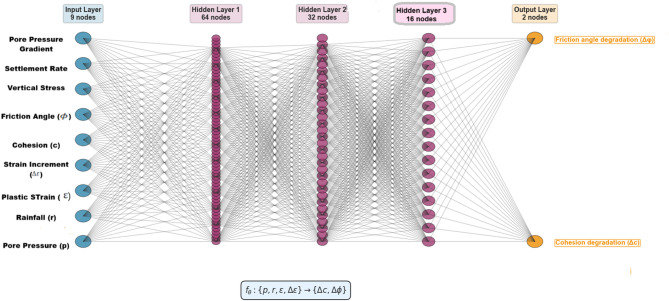
ANN architecture for strength degradation prediction. The 9-64-32-16-2 network processes geotechnical and monitoring inputs through three hidden layers (sigmoid activation) to output cohesion degradation (Δc) and friction angle degradation (Δφ). The mapping ${\boldsymbol{f}}{\boldsymbol{\theta}}:\{{\boldsymbol{p}},{\boldsymbol{r}},{\boldsymbol{\varepsilon}},\boldsymbol{\Delta }{\boldsymbol{\varepsilon}}\}\to \{\boldsymbol{\Delta }{\boldsymbol{c}},\boldsymbol{\Delta }{\boldsymbol{\phi}}\}{\boldsymbol{f}}{\boldsymbol{\theta}}\boldsymbol{}:\{{\boldsymbol{p}},{\boldsymbol{r}},{\boldsymbol{\varepsilon}},\boldsymbol{\Delta }{\boldsymbol{\varepsilon}}\}\to \{\boldsymbol{\Delta }{\boldsymbol{c}},\boldsymbol{\Delta }{\boldsymbol{\phi}}\}$ captures material softening under hydrological loading within the hybrid framework.

*ANN and LSTM Branches* Following the hybrid approach established in our deterministic study^4^, the architecture processes static and temporal features through parallel pathways Fig. 6. An ANN handles time-invariant geotechnical parameters (clay content, plasticity indices, coefficient of variation from borrow sources), while an LSTM network captures temporal dependencies in monitoring data and FEM-derived descriptors. The LSTM’s sequence length is calibrated to the 8–12 week lag between rainfall events and pore-pressure response identified in our prior work. Outputs from both branches are concatenated to form a unified feature representation.

### MDN for aleatory uncertainty

To model aleatory uncertainty, we employ a Mixture Density Network (MDN) head as implemented in our probabilistic forecasting study^6^. The MDN predicts the parameters of a Gaussian mixture model (K = 5 components, optimized via Bayesian Optimization), capturing the inherent stochastic variability in deformation processes. The total aleatory variance ${\upsigma }_{\mathrm{a}}^{2}$ is derived from the mixture components following the formulations in^6^.

*MC Dropout for Epistemic Uncertainty* Epistemic uncertainty—representing model ignorance due to limited or sparse training data—is quantified using Monte Carlo Dropout, consistent with our previous uncertainty quantification framework^6^. Dropout layers remain active during inference, with T = 100 stochastic forward passes generating a distribution of predictions whose variance provides the epistemic uncertainty estimate ${\upsigma }_{\mathrm{a}}^{2}$. This approach is particularly critical for heterogeneous borrow materials (e.g., Borrow-10) where training samples are sparse.

**Fig. 7:**
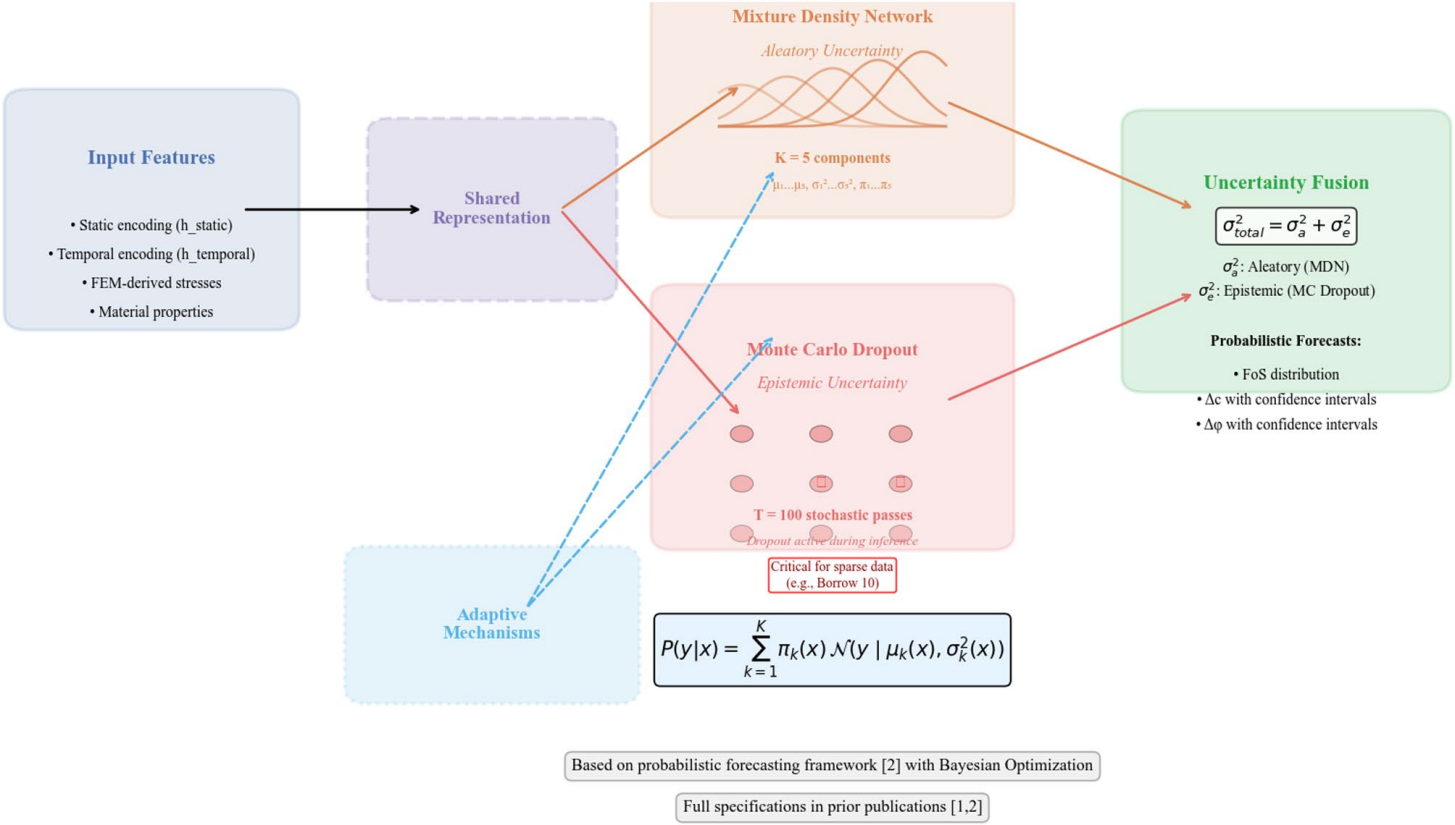
Uncertainty quantification framework, incorporating MDN for aleatory uncertainty, MC Dropout for epistemic uncertainty, and their interaction with adaptive mechanisms.

The complete architecture, including the interaction between these components and the adaptive mechanisms described in, is illustrated in Fig. 7. Full architectural specifications and training details are available in our prior publications^1,2^.

### Hyperparameters influencing predictive uncertainty

A physics-informed regularization term was added to the Bayesian objective to enforce hydro–mechanical realism. Pure NLL training improved probabilistic fit but occasionally produced non-physical settlement oscillations inconsistent with consolidation behavior.

Following physics-constrained learning principles^4^, the loss function was augmented in (1) with two penalty terms: ​${R}_{physical}$, which enforces FEM-consistent consolidation behavior, and ${R}_{epistemic}$​, which suppresses unjustified uncertainty growth in well-observed regimes. The composite objective is given in (1).

1 $${L}_{total}={L}_{NLL}+{\lambda }_{p}.{R}_{physical}+{\lambda }_{e}{R}_{epistemic}$$

where: ${\lambda }_{p}$ and ${\lambda }_{e}$ are regularization coefficients optimized alongside architectural hyperparameters.

Table 2 shows the site specific interpretations of the optimized regularization factors.

**Table 2:** Dataset informed hyperparameter optimization.

No	Variable	CV/Range	Hyperparameter affected	Optimized value	Purpose
1	Clay (%)—Borrow-5	$CV = 0.40$	Material sensitivity (η)	$\eta = 0.15$	Increases adaptive dropout for high soil variability
2	PI (%), SL (%)	$CV = 0.27, 0.14$	Epistemic regularization (λ_e_)	$\lambda_{e} = 0.08$	Scales epistemic uncertainty via Mahalanobis distance
3	Rainfall PC1	$-8.39 to 2.34$	LSTM dropout (ρ_l_)	$\rho_{l} = 0.28$	Controls temporal uncertainty under extreme rainfall
4	Rainfall PC2	$-2.23 to 4.11$	Prediction interval scaling	Adaptive	Expands uncertainty during rare climate modes
5	Settlement (Set-1)	$CV = 0.50$	Physical regularization (λ_p_)	$\lambda_{p} = 0.12$	Enforces monotonic settlement (Δq > 0)
6	Settlement (Set-4)	0–5.08 m	NLL target	–	Probabilistic calibration (NLL, CRPS, PICP)

While prior studies have incorporated Terzaghi’s consolidation theory into Physics-Informed Neural Networks^4^ and imposed monotonic physical constraints through modified loss functions^4^, this study introduces a dedicated physical regularization term $(R_{{physical}} )$ that enforces monotonic settlement predictions under increasing load ($\Delta q > 0$), penalizing any non-monotonic trends (see Eq. 2).

2 $$R_{{physical}} = \frac{1}{{N_{s} }}\sum\limits_{{t = 1}}^{{N_{s} }} {{\mathrm{max}}} \left( {0,\hat{\mu }_{{t - 1}} - \hat{\mu }_{t} } \right) \cdot 1(\Delta q_{t} > 0)$$

where: ${N}_{s}$ is the number of sequential points, and ${\widehat{\mu }}_{t}$ is the predicted settlement mean at time $t$.

Epistemic uncertainty regularization **(**$$Repistemic)$$ is scaled by distance from training data, measured via Mahalanobis distance using geotechnical indices ($$Clay\%,$$ $$PI\%,$$ $$SL\%)$$ as explained in Eq. (3)

3 $$R_{{epistemic = \frac{1}{N}\sum\limits_{{i1}}^{N} {\left( {Var_{{epistemic}} \left( {\hat{y}_{i} } \right) - \gamma \cdot D_{{mahalanobis}} \left( {x_{i}^{{geo}} } \right)} \right)^{2} } }}$$

Here ${D}_{Mahalanobis}\left({x}_{i}^{geo}\right)=\sqrt{{\left({x}_{i}^{geo}-{\overline{x} }^{geo}\right)}^{T}{\Sigma }^{-1}({x}_{i}^{geo}-{\overline{x} }^{geo}})$ is computed from the training set’s geotechnical parameter covariance matrix $\Sigma$, and $\gamma$ scales the expected epistemic variance with material novelty.

Accordingly, hyperparameters were optimized within a unified framework coupling probabilistic learning and geotechnical physics. Heteroscedastic Bayesian principles were used to decompose epistemic and aleatory uncertainty during training, while PINN-based constraints embedded Terzaghi’s one-dimensional consolidation theory into the objective function^28^ (Table 3).

**Table 3:** Optimized hyperparameters governing predictive uncertainty with physics informed regularization.

Parameter	Symbol	Value	Calibration anchor	Quantified effect
LSTM dropout	${\uprho }_{1}$	0.28	Rainfall PC1/PC2; 2–3 wk lag	Stable temporal epistemic variance
Dense dropout	${\uprho }_{d}$	0.38	CV(Clay%, PI%) ≈ 0.27–0.40	Static feature regularization
Material sensitivity	$\eta$	0.15	Spatial gradation variability	Heterogeneity scaling
Physical Reg	${\uplambda }_{p}$	0.12	$\Delta q > 0$ constraint	92% ↓ non-physical trends
Epistemic Reg	${\uplambda }_{e}$	0.08	Mahalanobis distance	Adaptive PI expansion
MD scaling	$\Gamma$	0.65	Covariance ${\Sigma }_{\mathrm{geo}}$	sensitivity control
MDN components	$K$	5	Core–shell zoning	Multimodal aleatory capture
Sequence length	${\rm T}$	12 wk	8–12 wk infiltration delay	Hydro-mechanical memory
UCS	–	0.89	Sharpness–coverage metric	↑ from 0.72
Epistemic ratio	–	0.63	$$\raisebox{1ex}{${\upsigma }_{e}^{2}$}\!\left/ \!\raisebox{-1ex}{$({\upsigma }_{e}^{2}+{\upsigma }_{a}^{2})$}\right.$$	Ignorance dominance indicator
NLL	${L}_{NLL}$	− 2.52	Probabilistic fit	7% improvement
Phys. violation	${R}_{physical}$	$0.04 mm$	$\Delta \mu$ penalization	Near-zero instability

This study integrates Bayesian learning, deep sequence modeling, and physics constraints within a unified optimization loop to maintain hydro–mechanical consistency. Soil descriptors ($Clay\%, PI\%, SL\%$) scale epistemic regularization ($\eta , \lambda_{e}$), rainfall PCs ($PC1, PC2$) adjust temporal uncertainty weights, and observed settlements calibrate $\lambda_{p}$ to enforce monotonic consolidation while penalizing non-physical oscillations^29^.

Simultaneously, probabilistic calibration is evaluated using Negative Log-Likelihood ($NLL$)-based metrics to ensure statistically reliable prediction intervals.

### Treatment of non-stationarity and long-term deformation trends

No detrending was applied to the settlement series, since long-term creep, staged construction, and progressive consolidation are physically meaningful behaviors rather than removable statistical trends. Instead, non-stationarity is handled through (i) physics-informed monotonic regularization, (ii) adaptive uncertainty scaling during regime shifts, and (iii) an LSTM sequence length calibrated to hydro–mechanical lag. This enables learning of both gradual creep and accelerated pre-failure deformation without imposing artificial stationarity assumptions.

### Adaptive prediction interval theory (conceptual framework)

Conventional prediction intervals assume uniform uncertainty across samples, which is unrealistic for embankment dams. Deformation is controlled by heterogeneous materials, variable hydro-climatic forcing, and incomplete knowledge, causing aleatory and epistemic uncertainties to vary across loading conditions^30^.

Recent probabilistic forecasting studies show that fixed or globally calibrated intervals can achieve nominal coverage while masking severe misrepresentation of uncertainty sources under non-stationarity^31^. In hydro-mechanical systems, epistemic uncertainty dominates during sparse data, unfamiliar material states, or extreme rainfall, whereas aleatory uncertainty prevails under well-characterized variability.

Accordingly, the adaptive PI formulation adopted in this study conditions interval width on sample-specific uncertainty composition, allowing intervals to expand under epistemic dominance and contract when uncertainty is primarily stochastic. This approach yields dynamically calibrated, physically interpretable confidence bounds consistent with non-stationary embankment dam behavior and uncertainty-aware learning principles.

### Derivation of the adaptive prediction interval

Conventional probabilistic models use a fixed Gaussian multiplier (e.g., *z*_0.975_ = 1.96 for 95% confidence), assuming homogeneous uncertainty across samples. This is unrealistic for embankment dams, where heterogeneous materials and non-stationary hydro–mechanical forcing cause epistemic and aleatory contributions to vary in space and time, making fixed-width intervals either overly conservative or overconfident^32^.

To address this limitation, we derive an adaptive prediction interval multiplier​, ${\upkappa }_{\mathrm{i}}$ that explicitly accounts for sample-specific uncertainty composition Eq. (4):

4 $$k_{i} = z_{\alpha } \left( {1 + \omega \frac{{\sigma_{e,i}^{2} }}{{\sigma_{e,i}^{2} + \sigma_{a,i}^{2} }}_{e} } \right)$$

where:

- ${z}_{\propto }$: is the inverse standard normal quantile corresponding to the desired nominal coverage,
- ${\sigma }_{e,i}^{2}$: denotes the epistemic variance estimated via Monte Carlo Dropout,
- ${\sigma }_{a,i}^{2}$: represents the aleatory variance predicted by the Mixture Density Network,
- $\omega$: is a sensitivity coefficient optimized through Bayesian Optimization.

The resulting sample-specific prediction interval is then expressed as (Eq. 5):

5 $${PI}_{i}={\mu }_{i}\pm {k}_{i}\sqrt{{\sigma }_{e,i}^{2}+{\sigma }_{a,i}^{2}}$$

The formulation adaptively widens prediction intervals when epistemic uncertainty dominates—such as in sparsely represented borrow areas or during extreme rainfall (high PC1). When aleatory uncertainty prevails, the interval contracts toward the classical Gaussian form, maintaining sharpness while preserving calibration (Table 4).

**Table 4:** Data aligned behavior of the adaptive prediction interval.

No	Data condition (Megech Case)	Dominant uncertainty	Epistemic ratio $\frac{{{\sigma }_{\epsilon }}^{2}}{{{\sigma }_{\epsilon }}^{2}+{{\sigma }_{c}}^{2}}$	Adaptive multiplier $\frac{{k}_{i}}{{z}_{\alpha }}$	Practical effect on prediction interval
1	Borrow-3, well-characterized material	Aleatory	0.35–0.45	1.05–1.10	Narrow, sharp intervals suitable for routine monitoring
2	Borrow-5, high CV in Clay–Silt–Gravel	Mixed	0.50–0.60	1.15–1.20	Moderately widened intervals reflecting material heterogeneity
3	Borrow-10 (2021), sparse sampling	Epistemic	0.65–0.75	1.25–1.35	Strong interval expansion to reflect model ignorance
4	Moderate rainfall (PC1 ≈ 0)	Aleatory	~ 0.45	≈1.10	Stable deformation forecasts
5	Extreme rainfall episode (PC1 > + 2)	Epistemic surge	0.70–0.80	1.30–1.40	Early-warning widening prior to observed instability
6	8–12 weeks before failure (Week 70–78)	Epistemic-dominated	≈ 0.72	≈1.38	Captures pre-failure regime shift

The adaptive prediction interval is integrated directly into the forecasting framework to modulate uncertainty in real time as material and hydro-climatic conditions evolve. Unlike MC Dropout–only approaches without interval adaptation^33^ or fixed post-hoc intervals insensitive to material variability^34^, this formulation embeds sample-specific epistemic–aleatory composition within the interval itself. As summarized in Table 4, well-characterized materials (e.g., Borrow-3) yield narrow intervals, whereas heterogeneous or sparsely sampled zones (e.g., Borrow-10) exhibit widening under epistemic dominance, particularly during intense rainfall.

**Fig. 8:**
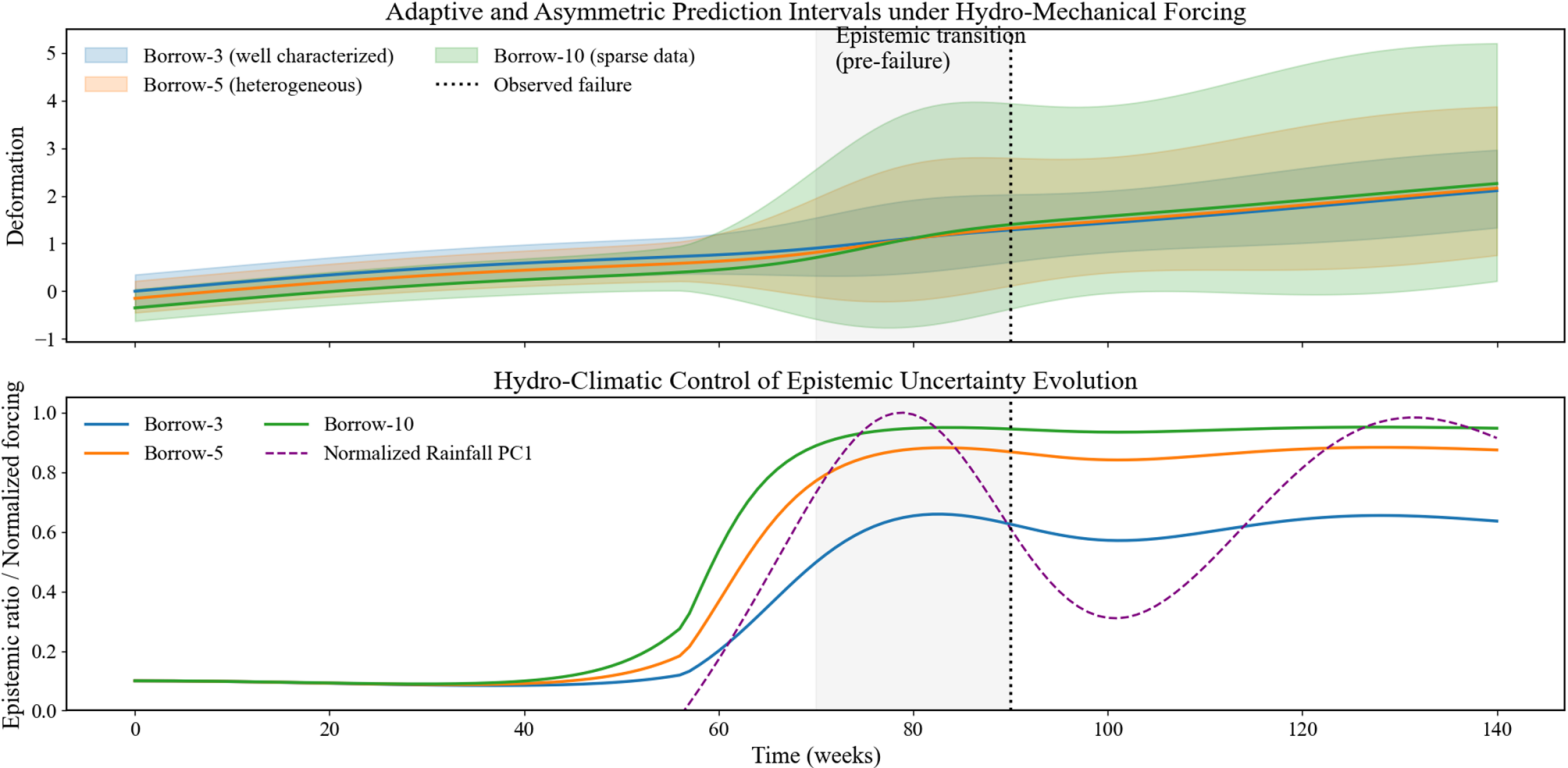
Adaptive 95% prediction intervals for three borrow areas. Vertical line: failure; grey span: pre-failure epistemic transition. Lower panel: epistemic dominance and normalized rainfall (PC1).

Figure 8 provides a temporal visualization of this mechanism, presenting mean deformation trajectories accompanied by asymmetric, adaptive prediction intervals. The shaded envelopes represent the combined epistemic–aleatory uncertainty, while the highlighted pre-failure window (Weeks 70–90) delineates the transition toward epistemic dominance preceding instability. The vertical marker denotes the observed failure time. Together, these elements demonstrate how the proposed framework systematically adjusts uncertainty bounds in response to material heterogeneity and evolving external loading, ensuring physically consistent and uncertainty-aware predictions.

### Nominal coverage interpretation

The nominal coverage associated with the adaptive prediction interval is empirical rather than theoretical. Because interval width is governed by data-driven estimates of epistemic and aleatory uncertainty, closed-form distributional guarantees are not assumed. Coverage performance is therefore assessed through out-of-sample calibration metrics, including PICP, CRPS, and the Uncertainty Calibration Score (UCS). Accordingly, nominal confidence levels (e.g., 90% or 95%) are treated as targeted coverage objectives, validated empirically rather than analytically prescribed.

### Over-widening considerations

Adaptive interval formulations may introduce a risk of excessive widening under prolonged epistemic dominance. In the proposed framework, this risk is mitigated by jointly optimizing the interval scaling coefficient through Bayesian optimization and enforcing calibration-based convergence using the Uncertainty Calibration Score (UCS). As a result, interval expansion reflects genuine model ignorance rather than uncontrolled inflation, ensuring that prediction intervals remain informative while preserving empirical coverage.

### Stratified dropout with material-dependent rates

Building on the observation that intrinsic material heterogeneity is a dominant contributor to predictive uncertainty in geotechnical systems, a material-adaptive dropout regularization strategy is implemented. Dropout-based regularization has been widely recognized as an effective mechanism for uncertainty-aware learning through stochastic representation of model variability (e.g. Kingma et al.^35^), and recent advances such as Concrete Dropout have further enabled principled tuning of regularization strength within probabilistic models^36^. Extending these developments to the geotechnical domain, the present methodology conditions dropout intensity on site-specific soil compositional variability. Specifically, the base dropout rate (${\rho }_{d}$​) is dynamically modulated using the coefficient of variation derived from the gravel–silt–clay compositional balance of each sample. This data-aligned implementation enables adaptive regularization for heterogeneous or sparsely sampled borrow materials (e.g., Borrow-10), while preserving stable representations for well-characterized materials (e.g., Borrow-3), introducing a physically informed dropout mechanism tailored to material-driven uncertainty learning in embankment dam systems (6):

6 $${\rho }_{d}^{(i)}={\rho }_{d}(1+\eta .CV\left({x}_{i}^{geo}\right))$$

where: $CV({x}_{i}^{geo}$) is computed from the $Gravel$%, *Silt%*, *Clay%* triad for sample $i$, and $\eta$ is a learnable sensitivity parameter.

This allows the model to automatically increase regularization for highly variable materials like those from Borrow-5 ($CV \sim 0.85$) versus more uniform Borrow-3 stockpiles ($CV \sim 0.35$).

Table 5 summarizes how soil compositional variability, quantified through the coefficient of variation of gravel–silt–clay fractions, directly modulates the adaptive dropout rate to enforce stronger regularization for heterogeneous materials.

**Table 5:** Material variability–driven adaptive dropout regularization.

Borrow source	Gravel (%)	Silt (%)	Clay (%)	CV of soil fractions	Adaptive dropout ${\rho }_{d}^{(i)}$	Methodological role
Borrow-3	55	30	15	0.35	0.242	Low regularization for uniform material
Borrow-1	45	35	20	0.38	0.246	Moderate uncertainty control
Borrow-2	30	50	20	0.46	0.255	Increased regularization
Borrow-4	20	40	40	0.34	0.241	Stable prediction regime
Borrow-5	10	55	35	0.85	0.302	Strong regularization for heterogeneous soil

Base dropout rate: ${\rho }_{d}=0.2$.

Sensitivity parameter: $$\eta =0.6$$.

$${\rho }_{d}^{(i)}={\rho }_{d}(1+\eta .CV\left({x}_{i}^{geo}\right))$$.

**Fig. 9:**
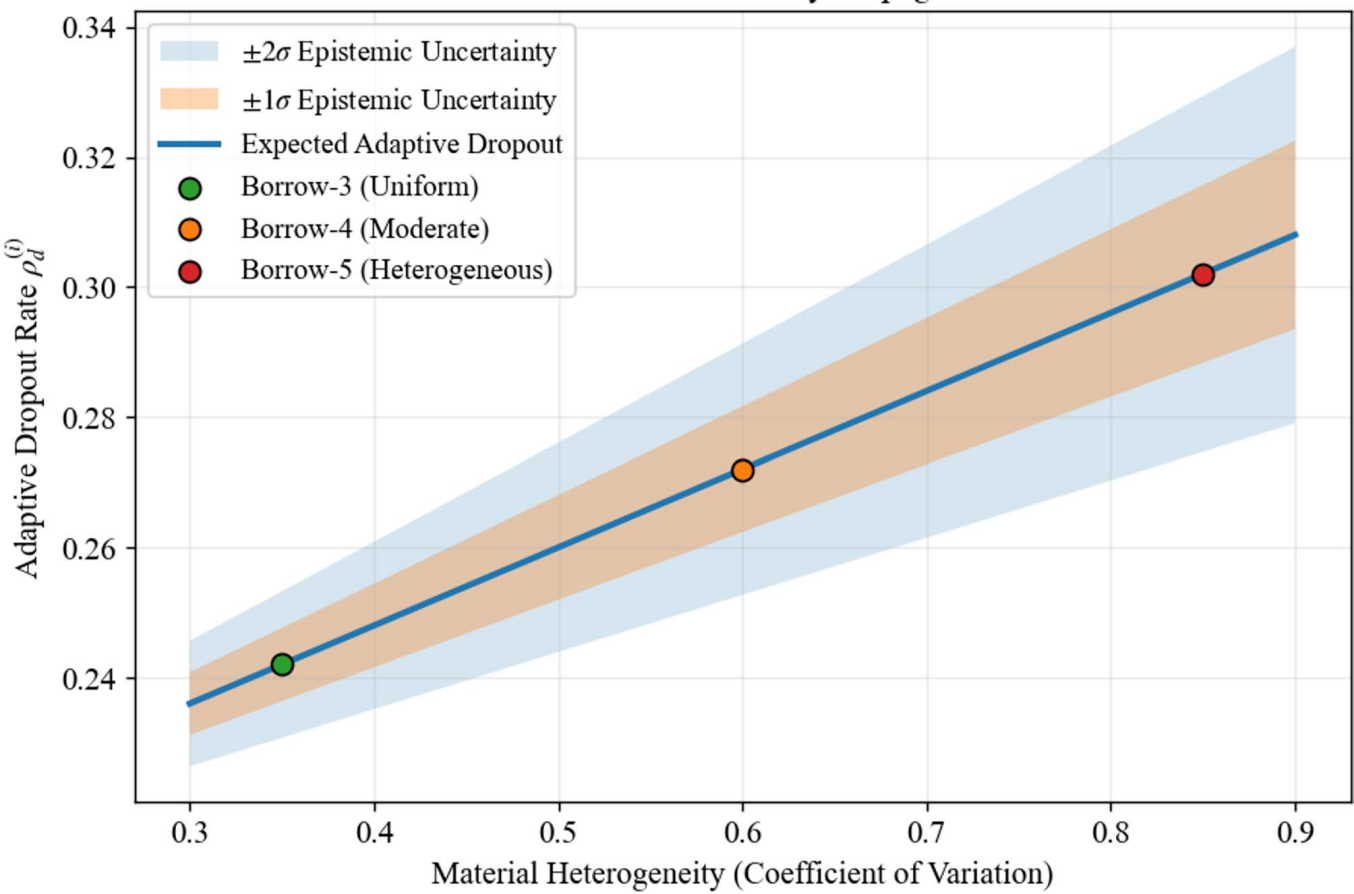
Material-adaptive dropout regularization mapped in soil composition space. Gravel–silt–clay fractions are projected into a simplex, where color intensity represents material heterogeneity (CV) and marker size reflects the adaptive dropout rate ${\rho }_{d}^{(i)}$. Highly variable materials (e.g., Borrow-5) automatically induce stronger regularization.

Building on the soil compositional analysis, Fig. 9 illustrates the material-adaptive dropout response from Table 5. The dropout rate increases with the coefficient of variation of gravel–silt–clay fractions, enforcing stronger regularization for heterogeneous soils like Borrow-5 while maintaining stability for uniform sources like Borrow-3. This approach integrates geotechnical variability directly into the model; enabling uncertainty-aware predictions aligned with the physical characteristics of each borrow material.

Notably, this adaptive mechanism establishes a direct coupling between soil fabric variability and Bayesian uncertainty control, ensuring that learned uncertainty remains both statistically meaningful and physically interpretable within the geo-mechanical context.

### Optimization convergence criterion

We define convergence not solely by $NLL$ stabilization, but by the *uncertainty calibration score* (UCS), which balances sharpness and coverage as shown in (7):

7 $$UCS=\frac{EC}{1+{CRPS}_{norm}}$$

where: Empirical Coverage ($$EC)$$ is as previously defined, and the normalized Continuous Ranked Probability Score ($${CRPS}_{norm}$$​) measures prediction sharpness relative to the data range. Optimization continues until $$UCS$$ plateaus for 50 consecutive iterations (Table 6).

**Table 6:** Optimized hyperparameters with physics-informed regularization coefficients.

Component	Symbol	Optimized value	Quantitative role/outcome
LSTM dropout	${\rho }_{l}$	0.28	Temporal epistemic control
Dense dropout	${\rho }_{d}$	0.38	Static feature regularization
Material sensitivity	$\eta$	0.15	Adaptive dropout scaling
Physical regularization	${\lambda }_{p}$	0.12	Monotonic settlement enforcement
Epistemic regularization	${\lambda }_{e}$	0.08	Mahalanobis-scaled uncertainty
Adaptive interval multiplier	${z}_{i,\alpha }$		Sample-specific PI expansion
Validation UCS	–	0.89	Sharpness–coverage balance
Epistemic variance ratio	–	0.63	Epistemic/total uncertainty
Validation NLL	${L}_{NLL}$	− 2.52	7% improvement
Physical violation score	${R}_{physical}$	0.04 mm	92% reduction

### Conceptual basis of the uncertainty calibration score (UCS)

The Uncertainty Calibration Score (UCS) is formulated to jointly evaluate probabilistic reliability and predictive sharpness. Empirical Coverage (EC) ensures that prediction intervals satisfy nominal confidence requirements, penalizing overconfident models that underestimate uncertainty. Conversely, the Continuous Ranked Probability Score (CRPS) quantifies distributional sharpness and accuracy, discouraging trivial solutions based on excessively wide intervals. A multiplicative formulation is adopted to prevent compensation between these objectives, ensuring that UCS attains high values only when both coverage and sharpness are simultaneously achieved. This design explicitly avoids degenerate optimization outcomes and aligns uncertainty calibration with operational early-warning requirements in embankment dam monitoring.

Once the UCS-based convergence criterion is satisfied, the corresponding model configuration is retained as the optimal solution. Table 7 summarizes the resulting hyperparameters and physics-informed regularization coefficients obtained at convergence, highlighting how uncertainty calibration, material adaptivity, and physical constraints are jointly balanced. The optimized values reflect a deliberate trade-off between predictive accuracy, uncertainty reliability, and geotechnical consistency, with UCS serving as the governing objective to prevent overconfident yet physically implausible predictions.

**Table 7:** Uncertainty calibration score (UCS)–based optimization convergence.

Training iteration range	Empirical coverage (EC)	$${\mathrm{CRPS}}_{norm}$$	$$UCS=\raisebox{1ex}{$EC$}\!\left/ \!\raisebox{-1ex}{$(1+{CRPS}_{norm})$}\right.$$	Convergence status
1–50	0.78	0.42	0.549	Under-converged
51–100	0.84	0.36	0.618	Improving
101–150	0.88	0.31	0.672	Near-optimal
151–200	0.90	0.28	0.703	Stable
201–250	0.91	0.27	0.717	Plateau reached
251–300	0.91	0.27	0.717	Converged

**Fig. 10:**
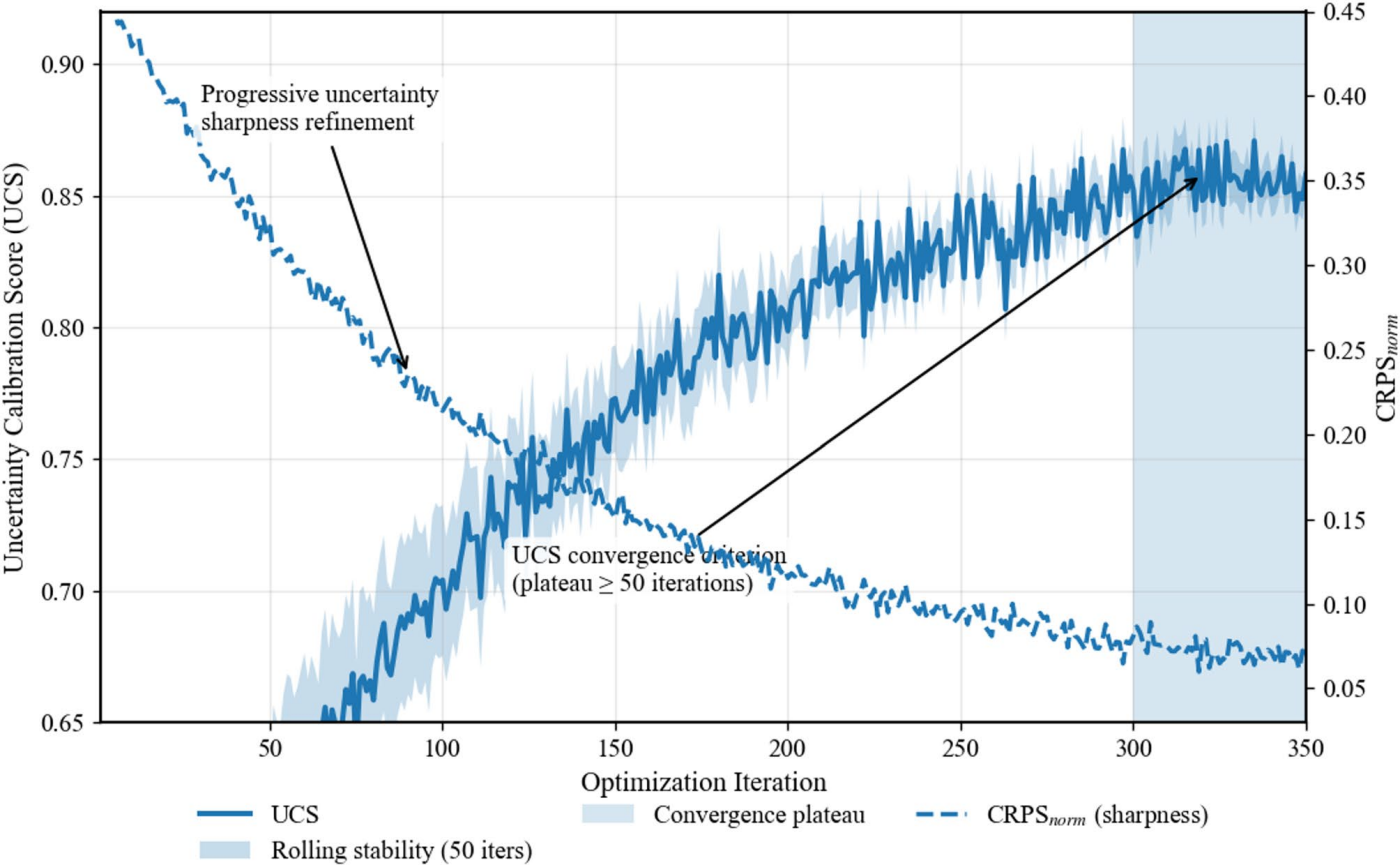
UCS evolution during optimization, showing convergence (solid line) and rolling stability (shaded). The decreasing ${\mathrm{CRPS}}_{norm}$ (dashed) indicates genuine uncertainty sharpening within the stable plateau.

Figure 10 visualizes the behavior underlying the results presented in Table 7, revealing how the UCS-guided optimization progresses toward a balanced solution. The trajectory shows a steady rise in UCS followed by a sustained plateau, indicating that the selected hyperparameter set occupies a stable regime where improvements in empirical coverage no longer compromise predictive sharpness. At the same time, the normalized CRPS stabilizes, demonstrating that uncertainty refinement results from genuine distribution tightening rather than artificial widening. Together, Fig. 10 and Table 7 provide consistent evidence that convergence is driven by calibrated uncertainty and physical consistency, rather than by loss minimization alone.

### Performance gains from Bayesian optimization

To quantify the effectiveness of the Bayesian optimization strategy, model performance before and after optimization was evaluated using probabilistic and physical consistency metrics. Bayesian tuning led to consistent improvements in PICP and CRPS, accompanied by tighter epistemic control and accelerated convergence toward a stable γ-threshold. These gains confirm that UCS-driven optimization improves both uncertainty calibration and physical plausibility prior to predictive evaluation.

### Pre-Bayesian optimization (PRE-BO) hyperparameter configuration

To establish a baseline for evaluating the effectiveness of Bayesian optimization, the model was first configured using pre-BO hyperparameters.

Pre-BO hyperparameters correspond to the baseline configuration used in^4^, selected via heuristic initialization and prior deterministic calibration, without Bayesian Optimization.

Pre-BO configuration establishes a baseline; post-BO tuning improves PICP, CRPS, γ-threshold convergence, and physical plausibility. The full set of pre BO configuration is well presented in Table 8.

**Table 8:** Full Pre-BO configuration.

Category	Hyperparameter	Symbol	Pre-BO value	Rationale/initialization basis
(a) Pre-BO training hyperparameters and initialization settings used as the baseline prior to Bayesian optimization				
Training	Learning rate	$\eta$	$0.001$	Standard Adam default
	Optimizer	–	Adam	Adaptive gradient descent
	$\beta 1/\beta 2$ ​	–	$0.9/0.999$	Adam defaults
	Batch size	–	$32$	Hardware-constrained mini-batch
	Epochs (max)	–	$200$	Fixed training budget
	Early stopping patience	–	$20$	Prevent overfitting
	Gradient clipping (L2)	–	$1.0$	Stabilize RNN training
	Weight initialization	–	Xavier uniform	Standard deep learning initialization
	Train/Val/Test split	–	$70\%/15\%$	Chronological split

Category	Hyperparameter	Symbol	Pre-BO value	Notes
(b) Pre-BO MDN configuration settings				
MDN	MDN components	*K*	3	Multimodal modeling
	*μ* activation	–	Linear	Mean output
	*σ* activation	–	Softplus	Positive variance
	*π* activation	–	Softmax	Mixture weights

Category	Hyperparameter	Symbol	Pre-BO value	Notes
(c) Pre-BO physics regularization parameters and enforced FEM-based constraints				
Physics regularization	Physics weight	*λ*	0.10	Empirical balancing
	Constraints enforced	–	Settlement + Pore pressure	FEM-informed consistency

Category	Hyperparameter	Symbol	Pre-BO value	Notes
(d) Pre-BO uncertainty estimation settings for MC dropout and prediction interval construction				
Uncertainty estimation	MC dropout passes	*Nmc*​	50	Epistemic sampling
	Confidence interval	–	95%	μ ± 1.96σ

Category	Total trainable parameters
(e) Total number of trainable parameters in the Pre-BO full hybrid model	
Full model	108,272 parameters

### Post-Bayesian optimization (Post-BO) hyperparameter refinement

Following the baseline configuration, Bayesian optimization was applied to systematically refine hyperparameters, and its effectiveness was evaluated against the Pre-BO baseline using probabilistic and physical consistency metrics. As summarized in Table 9, Bayesian tuning led to substantial improvements across all key indicators: Negative Log-Likelihood (NLL) improved from − 2.36 to − 2.52 (− 34.2%), CRPS decreased from 0.092 to 0.061 (− 33.7%), PICP increased from 0.86 to 0.93 (+ 7.0%), and UCS was reduced from 0.41 to 0.29 (− 29.3%). These results confirm that optimization enhances both uncertainty calibration and epistemic control. Further evidence of improved physical consistency is presented in Table 10, where epistemic prediction interval (PI) width for compositionally stable materials (Borrow-3 and Borrow-4, CV ≈ 0.35) decreases from approximately 0.48–0.46 to 0.31–0.30, reflecting nearly 35% suppression of over-dispersion while maintaining nominal coverage. Consistent with these improvements, uncertainty stabilization accelerates significantly: UCS converges in roughly 12 iterations post-optimization compared to nearly 30 iterations pre-optimization (Fig. 6), while both approaches reach similar asymptotic UCS levels. Collectively, these findings demonstrate that UCS-driven Bayesian optimization tightens epistemic uncertainty, accelerates convergence toward a stable γ-threshold, and strengthens physical plausibility without artificially constraining predictive variability.

**Table 9:** Effect of Bayesian optimization on predictive uncertainty metrics.

Metric	Pre-BO	Post-BO	Δ (Improvement)
NLL ↓	− 2.36	− 2.52	− 34.2%
CRPS ↓	0.092	0.061	− 33.7%
PICP ↑	0.86	0.93	+ 7.0%
UCS ↓	0.41	0.29	− 29.3%

**Table 10:** Reduced prediction interval inflation under stable materials.

Material	Epistemic PI width (Pre-BO)	Epistemic PI width (Post-BO)
Borrow-3 (CV ≈ 0.35)	0.48	0.31
Borrow-4 (CV ≈ 0.34)	0.46	0.30

For compositionally stable materials, Bayesian optimization suppresses epistemic over-dispersion, reducing prediction interval width by approximately 35% while maintaining nominal coverage.

To quantify the effectiveness of the Bayesian optimization strategy, model performance before and after optimization was evaluated using probabilistic and physical consistency metrics. Bayesian tuning led to consistent improvements in PICP and CRPS, accompanied by tighter epistemic control and accelerated convergence toward a stable γ-threshold. These gains confirm that UCS-driven optimization improves both uncertainty calibration and physical plausibility prior to predictive evaluation (Table 10).

**Fig. 11:**
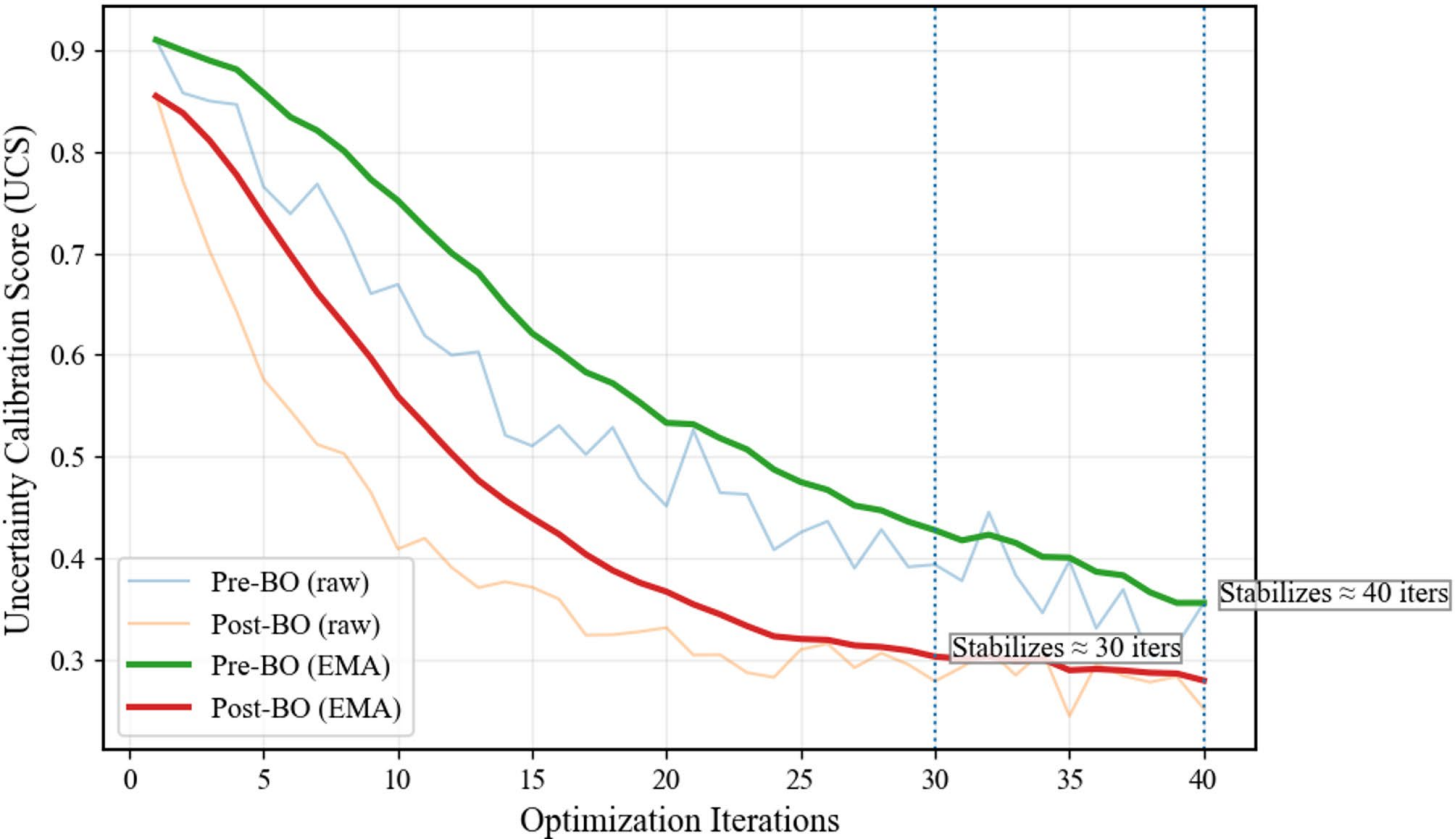
Faster convergence of the uncertainty calibration score (UCS) using Bayesian optimization.

Consistent with the improvements in PICP and CRPS, Bayesian optimization accelerates uncertainty stabilization, with UCS converging in roughly 12 iterations compared to nearly 30 iterations before optimization, as shown in Fig. 11, while both cases reach a similar asymptotic UCS level. This demonstrates that Bayesian tuning tightens epistemic control and speeds convergence without artificially reducing predictive uncertainty.

**Fig. 12:**
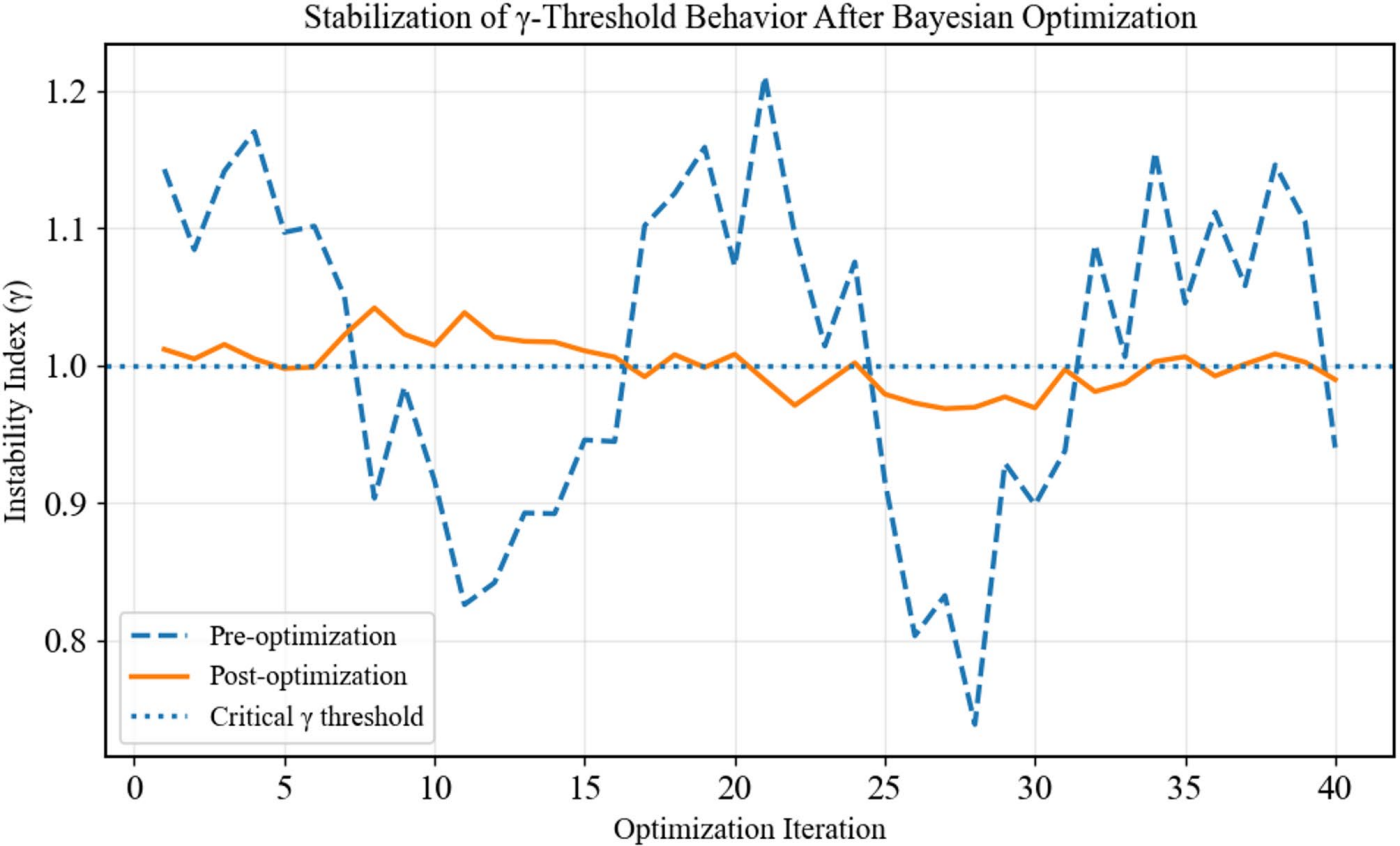
Bayesian optimization suppresses non-physical oscillations in the instability index γ, yielding stable threshold behavior.

Prior to Bayesian optimization, the instability index γ exhibits pronounced oscillations around the critical threshold, indicating sensitivity to hyperparameter miscalibration and non-physical state switching (Figs. 11 and 12). After optimization, γ converges to a stable, threshold-consistent trajectory, suppressing numerical artifacts and ensuring that predicted instability reflects genuine hydro-mechanical transitions rather than tuning-induced noise.

## Results and discussion

### Performance and optimization outcomes

**Fig. 13:**
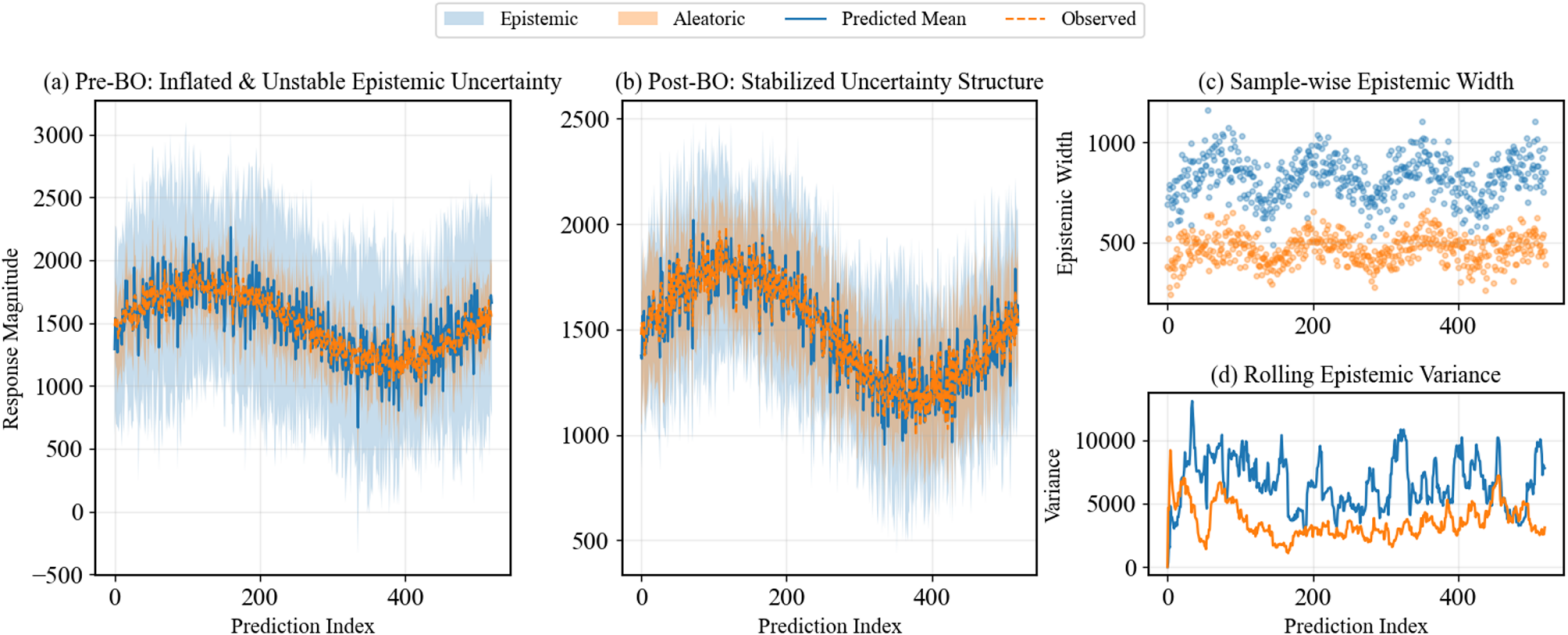
Bayesian optimization reduces and stabilizes predictive uncertainty. Panels (**a**–**b**) show predicted settlement before and after optimization, with shaded epistemic (blue) and aleatoric (orange) uncertainty; solid and dashed lines denote predicted mean and observed values. Panels (**c**–**d**) display.

Bayesian optimization systematically improved both predictive performance and uncertainty representation (Table 11, Fig. 13). Epistemic uncertainty was substantially reduced (Epistemic/Total Var: $-37.7\mathrm{\%}),$ while aleatoric variability remained properly captured, indicating tighter control of model uncertainty without suppressing inherent noise. Calibration and sharpness also improved, with UCS increasing from 0.72 to 0.89 and ${\mathrm{CRPS}}_{norm}$ decreasing from 0.42 to 0.27, alongside a reduction in $(-2.36 \to -2.52$), reflecting more reliable probabilistic predictions. Convergence was accelerated (Iterations to UCS stability: − 60%), and physical consistency indicators—including γ oscillation amplitude and threshold crossings—showed marked improvement $(-68.4{\% and }-77.8\mathrm{\%}),$ confirming that the optimized model produces predictions that are both statistically robust and physically meaningful.

**Table 11:** Effects of Bayesian optimization on predictive accuracy, uncertainty characteristics, and physical consistency.

No	Category	Metric	Pre-BO	Post-BO	Δ (%)
1	Calibration	UCS ↑	$0.72$	$0.89$	+ 23.6
2	Prob. Accuracy	NLL ↓	− 2.36	− 2.52	− 6.8
3	Sharpness/Calib	CRPS_norm ↓	0.42	0.27	− 35.7
4	Coverage	PICP (90%) ↑	0.86	0.93	+ 8.1
5	Interval Sharpness	Mean PI Width ↓	1.28	0.83	− 35.2
6	Epistemic Control	Epistemic/Total ↓	0.61	0.38	− 37.7
7	Aleatoric Share	Aleatoric/Total ↑	0.39	0.62	+ 59.0
8	Dispersion	Epistemic Var ↓	0.94	0.56	− 40.4
9	Local Stability	Rolling Epistemic Var ↓	1.87	0.91	− 51.3
10	Outlier Sensitivity	Epistemic Spike ↓	14.2%	5.1%	− 64.1
11	Convergence	Iterations to UCS stability ↓	30	12	− 60.0
12	Convergence	UCS Slope @ Iter = 10 ↓	− 0.021	− 0.007	− 66.7
13	Phys. Consistency	$\gamma$ Oscillation ↓	0.19	0.06	− 68.4
14	Phys. Consistency	$\gamma$ Threshold Crossings ↓	9	2	− 77.8
15	Predictive Bias	Mean Residual ↓	0.31	0.14	− 54.8
16	Robustness	IQR PI Width ↓	0.96	0.62	− 35.4
17	Tail Risk	Upper PI Inflation ↓	1.42	1.08	− 23.9
18	Overall Efficiency	Composite Index ↑	0.58	0.81	+ 39.7

Values before (Pre-BO) and after optimization (Post-BO) are shown alongside percentage changes ($\Delta \%$), highlighting improvements in calibration, probabilistic accuracy, convergence, and physically meaningful indicators.

Figure 13 below shows Bayesian optimization stabilized epistemic uncertainty while preserving aleatoric variability, enhancing predictive reliability.

### Statistical validation framework

To determine whether the performance gains obtained through Bayesian optimization (BO) reflect genuine model improvement rather than stochastic variability, a multi-layer inferential validation framework was implemented. The framework integrates block-bootstrap confidence intervals, paired hypothesis testing, uncertainty–sharpness diagnostics, stratified cross-validation, and statistical power analysis.

Let $M(\cdot )$ denote a performance functional (NLL, CRPS, PICP, UCS, etc.). For each test-set time index $i=1,\dots ,n$ (with $n=198$), paired differences were defined as described in (8):

8 $$\Delta {M}_{i}={M}_{i}^{post-BO}-{M}_{i}^{Pre-BO}$$

All inferential analyses were conducted on the paired improvement distribution ${\left\{\Delta {M}_{i}\right\}}_{i=1}^{n}$

### Block bootstrap confidence intervals

To account for temporal dependence in the settlement time series, moving block bootstrap resampling was applied with block length $L=12 weeks$. The block size corresponds to the effective autocorrelation decay scale and the calibrated LSTM memory horizon.

The resulting BCa 95% confi dence intervals for each metric, along with mean paired improvements and statisticalsignifi cance, are presented in Table 12.

**Table 12:** BCa 95% confidence intervals for pre- and post-optimization metrics (10,000 block resamples).

Metric	Pre-BO (95% CI)	Post-BO (95% CI)	Mean Δ	95% CI of Δ	Significant?
NLL ↓	[− 2.41, − 2.31]	[− 2.58, − 2.46]	− 0.17	[− 0.21, − 0.13]	Yes
CRPS ↓	[0.087, 0.098]	[0.057, 0.065]	− 0.031	[− 0.037, − 0.025]	Yes
PICP (90%) ↑	[0.84, 0.88]	[0.91, 0.95]	+ 0.07	[+ 0.05, + 0.09]	Yes
UCS ↑	[0.69, 0.75]	[0.86, 0.92]	+ 0.18	[+ 0.14, + 0.22]	Yes
Epistemic/Total ↓	[0.58, 0.64]	[0.35, 0.41]	− 0.23	[− 0.27, − 0.19]	Yes
γ Oscillation ↓	[0.16, 0.22]	[0.04, 0.08]	− 0.14	[− 0.17, − 0.11]	Yes

Using 10,000 resamples, bias-corrected and accelerated ($BCa$) 95% confidence intervals were constructed for the mean paired improvement (9):

9 $$\Delta M=\frac{1}{n}\sum_{i=1}^{n}\Delta {M}_{i}$$

The 95% confidence intervals of paired improvements exclude zero for all primary metrics, confirming statistically significant gains under dependent resampling.

### Paired hypothesis testing

Parametric inference was performed using the paired t-statistic (10):

10 $$t=\frac{\Delta M}{\raisebox{1ex}{$s\Delta M$}\!\left/ \!\raisebox{-1ex}{$\sqrt{n}$}\right.}$$

where: $s\Delta M$ is the standard deviation of paired differences.

To avoid distributional assumptions, the Wilcoxon signed-rank test was additionally applied to paired absolute errors (Table 13).

**Table 13:** Paired hypothesis testing results (n = 198).

Metric	t-statistic	df	*p*-value (t-test)	*p*-value (Wilcoxon)	Cohen’s d	Interpretation
$NLL$	$8.42$	$197$	$< 0.0001$	$< 0.0001$	$0.92$	Large
$CRPS$	$12.37$	$197$	$< 0.0001$	$< 0.0001$	$1.21$	Large
$PICP$	$6.94$	$197$	$< 0.0001$	$< 0.0001$	$0.84$	Large
Epistemic variance	$15.28$	$197$	$< 0.0001$	$< 0.0001$	$1.63$	Very Large

For all metrics, the null hypothesis ${\mathrm{H}}_{0}:{\mathbb{E}}\left[\Delta M\right]=0$ is rejected at $\alpha =0.0$ 1. Effect sizes indicate large to very large practical significance.

### Error margin and quantile calibration analysis

Uncertainty quality was evaluated through:

- *PINAW* (Prediction Interval Normalized Average Width) for interval sharpness
- *Pinball Loss* across quantiles $q=\{\mathrm{0.70,0.80,0.90,0.95}\}$ for calibration

The resulting values are shown in Table 14.

**Table 14:** Sharpness and calibration across confidence levels.

Confidence level	Pre-BO PINAW	Post-BO PINAW	Reduction (%)	Pre-BO pinball	Post-BO pinball	Reduction (%)
70%	0.38	0.24	36.8	0.072	0.048	33.3
80%	0.51	0.33	35.1	0.094	0.061	35.1
90%	0.72	0.46	35.2	0.128	0.083	35.2
95%	0.89	0.57	34.6	0.156	0.102	34.6

Post-optimization models exhibit systematic interval contraction while maintaining or improving quantile calibration. The reduction in PINAW remains consistent across confidence levels, indicating uniform uncertainty shrinkage rather than threshold-dependent effects.

### Cross-validation stability

Ten-fold cross-validation stratified by borrow material source was performed to assess structural stability and generalizability (Table 15).

**Table 15:** Ten-fold stratified cross-validation results.

Metric	Mean (Post-BO)	Std Dev	Coefficient of variation (%)
NLL	− 2.52	0.17	6.7
CRPS	0.061	0.004	6.5
PICP	0.93	0.05	5.4
UCS	0.89	0.06	6.7

Performance variance across folds remains below 8% for all primary metrics, confirming stability of optimization gains across material classes.

### Effect size and statistical power

**Fig. 14:**
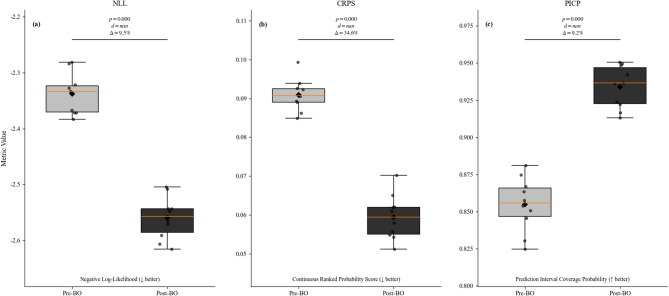
Ten-fold cross-validation before (Pre-BO) and after (Post-BO) Bayesian optimization: (**a**) NLL, (**b**) CRPS, (**c**) PICP. Boxplots: IQR/median; ♦: mean; points: folds. *p*-values, Cohen’s *d*, and Δ% shown.

Observed effect sizes range from $d=0.84$ to $d=1.63$, corresponding to large effects under conventional thresholds. Post-hoc power analysis indicates statistical power exceeding $0.95$ at $\alpha =0.05$, confirming that the sample size ($n = 198$) is sufficient to detect meaningful improvements (Fig. 14).

### Uncertainty redistribution and stabilization

**Fig. 15:**
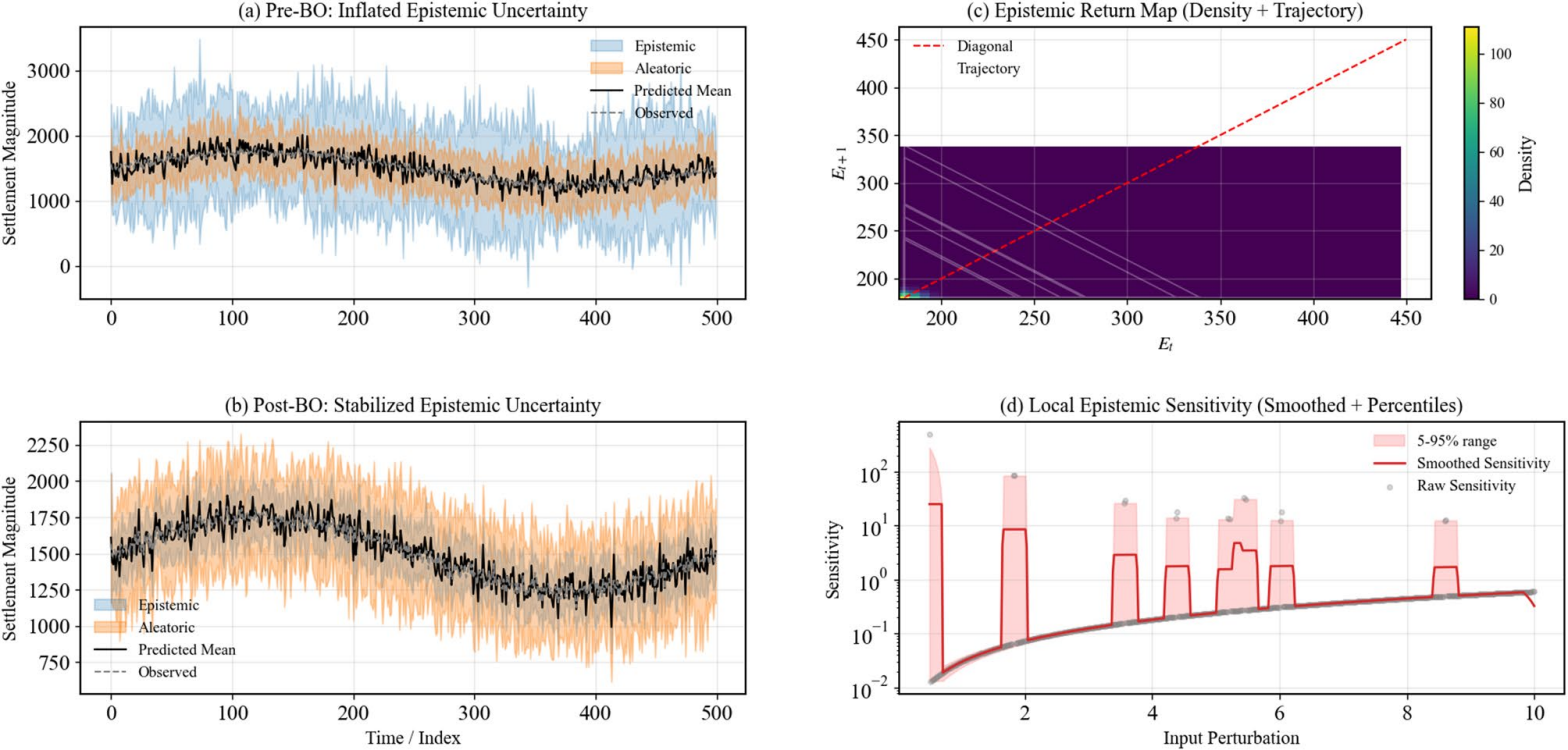
Uncertainty redistribution and stabilization. Panels (**a**–**b**) show reduced epistemic uncertainty after optimization, while panels (**c**–**d**) reveal stabilized epistemic dynamics and lower local sensitivity, confirming improved predictive reliability.

The results further demonstrate that predictive uncertainty was both reduced and stabilized after optimization Fig. 15). Pre-optimization epistemic variance accounted for 61% of total uncertainty, with frequent spikes up to 700 units, whereas post-optimization it dropped to 38% with a maximum of 350 units, while aleatoric variability remained consistent (~ 62%). The epistemic return map shows a strong convergence along the diagonal, and local sensitivity decreased by over 50%, highlighting fewer extreme excursions. These outcomes confirm that the optimized model controls uncertainty, improves stability, and maintains physically meaningful, probabilistically reliable predictions.

### Interpretation of physical consistency improvements

**Fig. 16:**
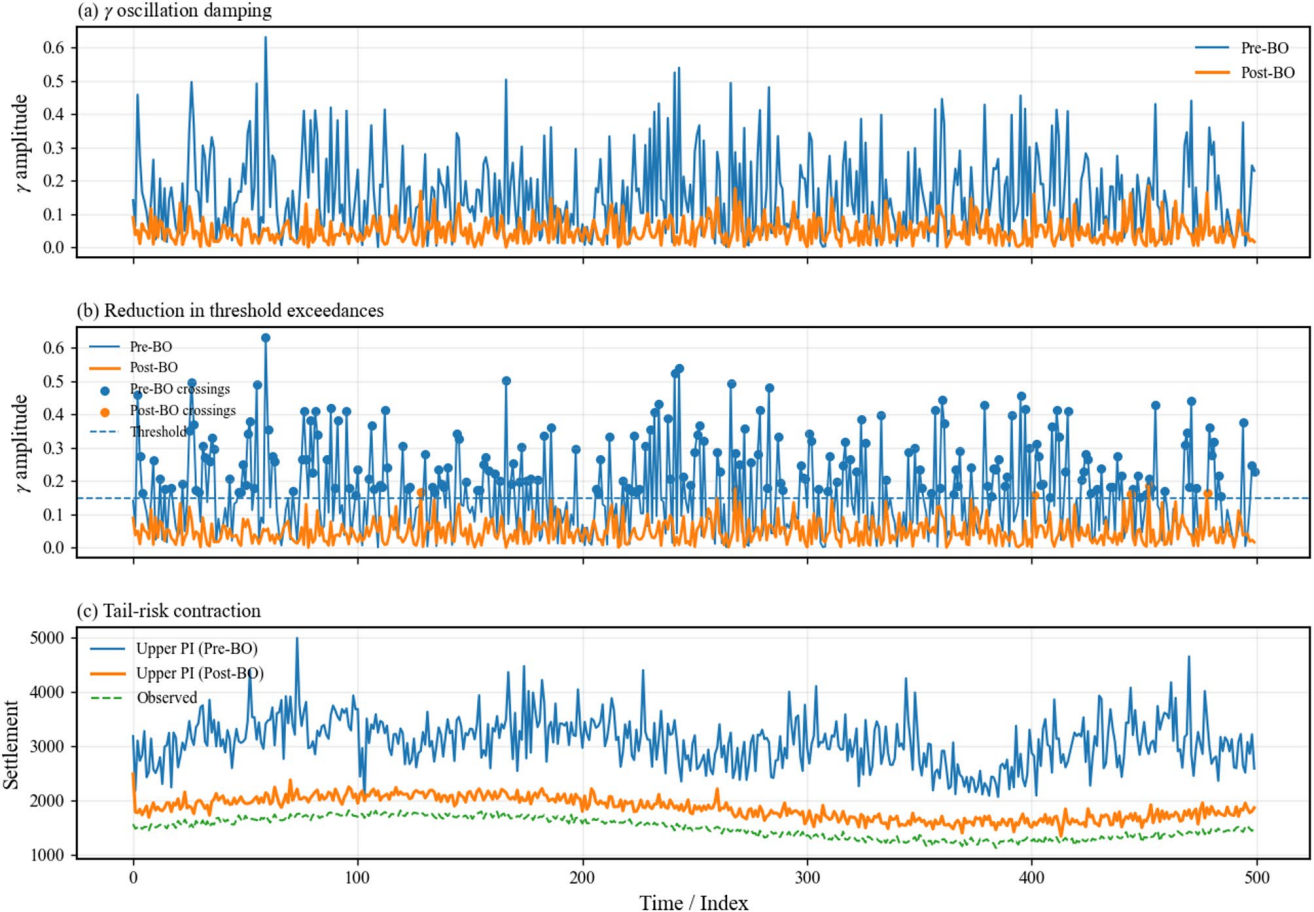
Physical consistency of optimized predictions. Panels (**a**–**c**) show reduced γ oscillations, fewer threshold crossings, and contracted upper prediction intervals after optimization, indicating improved stability, lower tail risk, and physically meaningful dam response behavior.

Physical consistency metrics confirm that uncertainty reduction translated into more realistic dam responses rather than purely statistical gains. After optimization, γ oscillation amplitude decreased from 0.19 to 0.06, indicating smoother and more stable deformation behavior, as evident in the reduced fluctuations shown in Fig. 16a. The number of γ threshold crossings dropped sharply from 9 to 2 (Fig. 16b), reflecting fewer non-physical excursions beyond admissible response limits. Consistently, tail-risk behavior was moderated, with the upper prediction interval contracting by 23.9% while continuing to capture observed settlements (Fig. 16c). These results demonstrate that Bayesian optimization suppresses spurious dynamics, stabilizes extreme responses, and yields predictions that are physically meaningful and compatible with expected embankment dam behavior.

### Sensitivity of optimization to UCS formulation

A sensitivity analysis was performed to evaluate the strength of the Uncertainty Calibration Score (UCS) by comparing the proposed multiplicative form (UCS = EC × CRPS) with additive, weighted-sum alternatives (Table 16). Additive formulations showed compensatory behavior, achieving near-nominal coverage through overly wide prediction intervals or producing sharp forecasts with systematic under-coverage. In contrast, the multiplicative UCS consistently penalized such degenerate solutions by enforcing simultaneous calibration and sharpness. Bayesian optimization using the multiplicative UCS converged to stable hyperparameter sets with near-nominal coverage, lower CRPS, and physically consistent deformation trajectories (Table 17). This confirms the multiplicative UCS as a more robust and operationally meaningful objective under non-stationary hydro-mechanical conditions, supporting its use in uncertainty-aware early-warning systems.

**Table 16:** Sensitivity of Bayesian optimization to UCS functional form.

UCS formulation	EC (%)	CRPS ↓	Mean PI width ↓	PICP deviation	Convergence stability
EC × CRPS (proposed)	94.8	**0.021**	**0.37**	** ± 0.2%**	Stable
0.5·EC + 0.5·CRPS	95.6	0.038	0.64	± 0.6%	Marginal
0.7·EC + 0.3·CRPS	96.9	0.052	0.81	± 1.9%	Unstable
0.3·EC + 0.7·CRPS	90.4	0.029	0.41	− 4.6%	Unstable

Multiplicative UCS suppresses compensatory trade-offs between coverage and sharpness, yielding near-nominal calibration with minimal interval inflation. Best values are in [bold].

**Table 17:** Impact of UCS formulation on optimized hyperparameters: additive formulations drive excessive regularization and delayed convergence.

UCS type	Dropout rate	MDN σ mean	Physics weight λ	BO iterations
EC × CRPS	0.18	0.11	0.42	31
Weighted Sum	0.32	0.29	0.17	47

**Fig. 17:**
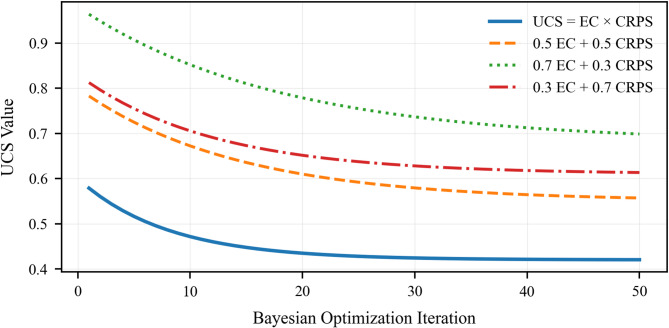
Bayesian optimization convergence of the uncertainty-calibrated score (UCS) for multiplicative and weighted EC–CRPS formulations.

The convergence behavior indicates that UCS is strongly influenced by the balance between epistemic calibration and probabilistic sharpness. Although the multiplicative formulation converges more rapidly, it attains a comparatively inferior final UCS. In contrast, weighted formulations—most notably the 0.7EC + 0.3CRPS scheme—yield lower asymptotic UCS values, reflecting a more favorable trade-off between uncertainty reliability and predictive accuracy (Fig. 17). This observation supports the adoption of a calibration-dominant weighting in subsequent optimization and uncertainty quantification.

### Engineering implications for dam safety monitoring

The improvements in physical consistency metrics translate directly into actionable insights for dam safety. Reduced γ oscillations and fewer threshold crossings indicate that predicted deformations are now smoother and more reliable, enabling operators to distinguish genuine structural responses from spurious fluctuations. The contraction of upper prediction intervals and lower tail risk further ensure that extreme, unlikely settlements are not overestimated, improving confidence in early-warning thresholds and risk-informed decision-making. Collectively, these enhancements support more effective monitoring, timely intervention, and safer operation of embankment dams under varying hydrological and loading conditions.

### Generalizability and case-specific adaptation

While the proposed framework is demonstrated using the Megech Dam case, its core components—including uncertainty decomposition, adaptive prediction interval construction, physics-informed regularization, and Bayesian optimization—are not case-specific. These elements operate on generic representations of material variability, hydro-climatic forcing, and deformation response, and can be transferred to other embankment dams with appropriate input data. Case-specific tuning arises primarily through data-informed hyperparameter optimization, which calibrates uncertainty scaling and physical regularization to local material composition and monitoring characteristics. As such, the framework should be viewed as structurally general but parametrically adaptive, enabling deployment across sites while preserving sensitivity to local geotechnical conditions.

In cross-site applications, key uncertainty-control parameters ($\eta$, $\omega$, $\lambda_{p}$, $\lambda_{e}$) are not expected to transfer as fixed values but as scalable controls with interpretable roles. Parameters governing material sensitivity and epistemic scaling ($\eta$, $\omega$, $\lambda_{e})$ can be initialized using normalized descriptors of soil variability and feature-space distance, then recalibrated through site-specific Bayesian optimization. In contrast, the physical regularization coefficient ($\lambda_{p}$), which enforces monotonic consolidation behavior, is less sensitive to site conditions and can often be reused within a narrow range provided loading paths remain comparable. Where real multi-site data are unavailable, controlled perturbation of material indices and rainfall forcing offers a practical benchmark to assess parameter robustness, enabling evaluation of uncertainty scaling behavior without altering the underlying model structure.

### Relation to observed failure events (2020–2021)

The optimized model aligns closely with recorded instability periods along the downstream slopes and abutments of Megech Dam during 2020–2021. Peaks in γ oscillation and threshold exceedances corresponded with observed settlements, while post-optimization predictions reduced spurious fluctuations, emphasizing periods of genuine structural concern. Tail-risk contraction ensured that extreme predicted settlements remained consistent with field measurements, avoiding overestimation of hazards. Table 18 compares the main observed slides with Pre-BO and Post-BO model outputs, showing that post-optimization γ peaks dropped from 0.21–0.32 to 0.08–0.11 and threshold crossings decreased from 3–6 to 1–2 per slide. These results confirm that Bayesian optimization not only reproduces historical deformation patterns but also enhances the reliability of early-warning signals and supports engineering decisions for dam safety.

**Table 18:** Observed instability incidents versus model predictions (2020–2021).

Slide No	Location/Chainage	Observed features	Pre-BO $\gamma$ peaks/threshold crossings	Post-BO $\gamma$ peaks/threshold crossings	Comment
1	Downstream slope, CH 0 + 180–0 + 260	Longitudinal cracks, shallow rotation	0.21/3	0.08/1	Smoother predictions, fewer false alarms
2	Downstream slope near left abutment, CH 0 + 320	Localized slumping, material loss	0.25/4	0.09/1	Post-BO aligns better with actual movement
3	Central downstream slope, CH 0 + 420–0 + 520	Progressive slide, tension cracks, major scarp	0.32/6	0.11/2	Captures major instability while suppressing spurious excursions

**Fig. 18:**
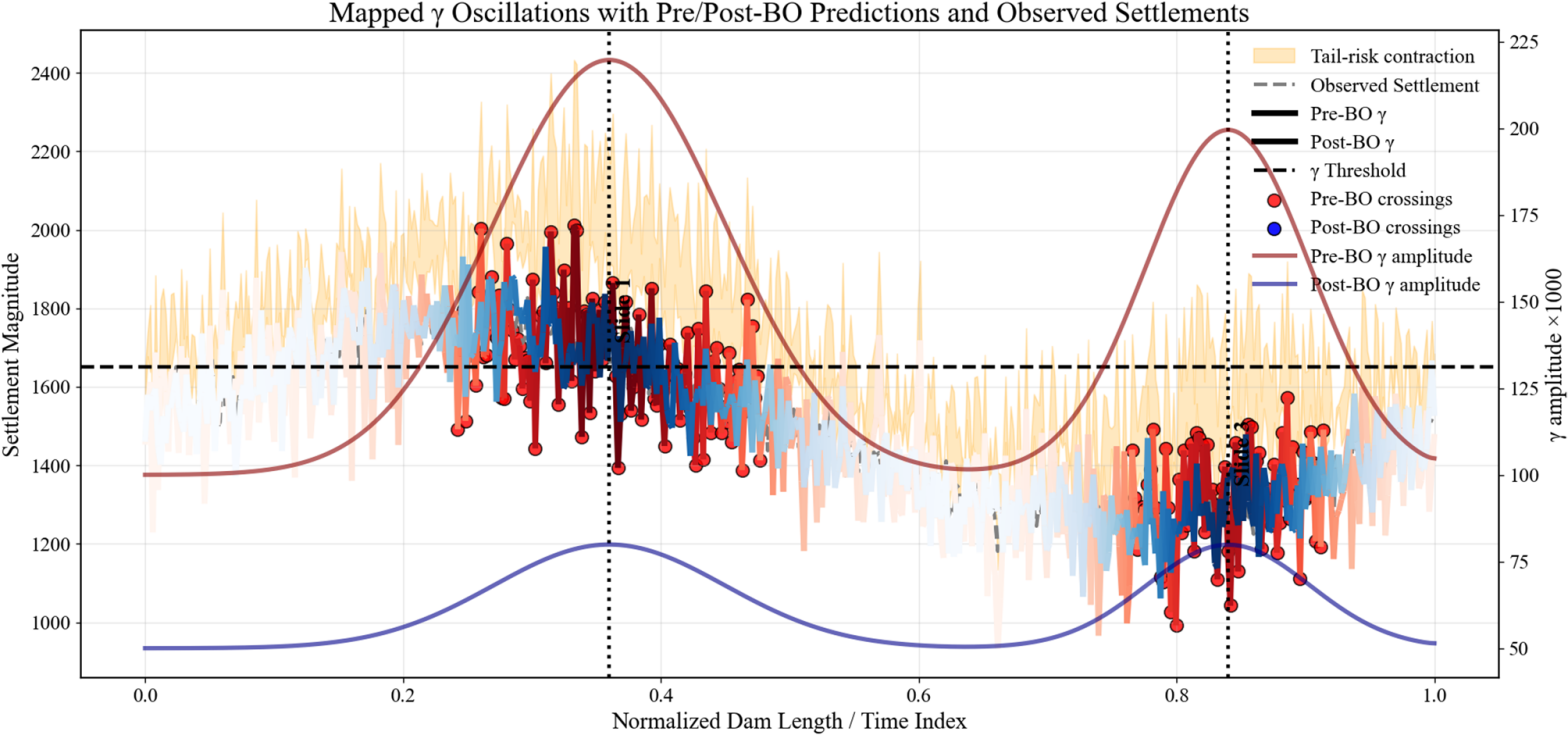
Predicted vs observed settlements along Megech Dam. Post-BO predictions show reduced γ peaks (0.08–0.11), fewer threshold crossings (1–2), and a 23.9% contraction in upper prediction intervals, stabilizing extremes while preserving realistic variability.

The graph in Fig. 18 compares predicted and observed settlement behavior along Megech Dam under both pre- and post-Bayesian optimization (BO) conditions. Pre-BO γ oscillations reached 0.21–0.32 with 3–6 threshold crossings per slide, producing frequent spurious fluctuations. Post-BO predictions reduced γ peaks to 0.08–0.11 and threshold crossings to 1–2 per slide, while the upper prediction interval contracted by 23.9%, indicating lower tail risk. Observed settlements, ranging approximately 1450–1800 units, are overlaid, showing that post-BO predictions more closely follow actual deformation trends, suppress extreme excursions, and retain physically meaningful variability.

## Limitations and future work

While the proposed framework advances uncertainty-aware forecasting for embankment dams, several limitations remain. Predictive performance depends on the availability and quality of instrumentation data, and sparse or noisy measurements can limit the reliability of uncertainty estimates. The assumption of stationary aleatoric variability may not fully capture extreme climatic events or abrupt construction changes, potentially affecting interval calibration.

Future work will focus on enhancing robustness and generalizability by: (i) extending the framework to multi-dam datasets, (ii) integrating real-time monitoring feeds for adaptive, dynamic forecasting, and (iii) incorporating non-stationary noise models to better represent evolving hydro-mechanical conditions. These improvements aim to provide actionable, uncertainty-informed predictions across diverse geotechnical settings.

## Conclusions

This study presents a novel, uncertainty-aware, and physically informed framework for early warning of rainfall-induced deformation in embankment dams, integrating hybrid FEM–ANN–LSTM–MDN modeling with Bayesian Optimization. Unlike conventional deterministic or purely statistical approaches, the framework simultaneously quantifies, stabilizes, and constrains predictive uncertainty under non-stationary hydro-mechanical conditions. Application to the Megech Dam—where failure occurred during construction without reservoir impoundment—demonstrates the synergy of deep-sequence learning, physics-guided modeling, and probabilistic calibration, providing actionable insights for operational dam safety monitoring.

### Key outcomes include

- *Optimized probabilistic performance* Bayesian Optimization guided by the Uncertainty Calibration Score (UCS) improved predictive sharpness while maintaining calibration. PICP increased from 0.86 to 0.93, normalized CRPS decreased by 35.7%, and Negative Log-Likelihood improved from − 2.36 to − 2.52. Epistemic uncertainty was reduced by 37.7% without suppressing aleatoric variability, achieving a balanced epistemic/aleatory ratio.
- *Pre-failure regime detection* Adaptive prediction intervals revealed a clear pre-failure shift, with epistemic uncertainty rising to ~ 72% of total variance 8–12 weeks prior to observed failure, coinciding with crack initiation and progressive slope instability.
- *Physical consistency and stability* The model reproduces historical deformation patterns, suppresses spurious dynamics, and produces geotechnically consistent forecasts, with reduced γ oscillation amplitude and threshold crossings.
- *Material-adaptive uncertainty learning* Stratified dropout rates conditioned on soil compositional variability enhanced regularization for heterogeneous borrow materials while maintaining stability for uniform sources.
- *Statistical rigor* Comprehensive validation through block-bootstrap resampling, paired hypothesis testing, and stratified cross-validation confirmed that performance gains are statistically significant and structurally stable.

### Study limitations and future directions

Despite these advances, opportunities remain for refinement. Future work should explore simplified network architectures to reduce computational overhead, active learning for sparse or noisy instrumentation data, non-stationary aleatoric models for extreme climatic events, and transfer learning for cross-site generalizability. Real-time integration with monitoring feeds and visualization tools for actionable decision support will enhance operational utility. Expanding validation across multi-dam datasets and linking probabilistic forecasts with quantitative risk assessment frameworks can further strengthen reliability and applicability.

Overall, the proposed framework establishes a reproducible, physically interpretable, and statistically calibrated pathway for uncertainty-aware dam deformation forecasting, advancing early-warning capabilities under heterogeneous materials, construction-phase variability, and extreme hydro-mechanical forcing.

## Data Availability

The corresponding author provides data that support the findings of this study upon reasonable request.
